# H‑cluster Intermediates and Catalytic Properties of Clostridium pasteurianum [FeFe]-Hydrogenase III

**DOI:** 10.1021/acs.biochem.5c00066

**Published:** 2025-05-13

**Authors:** Effie C. Kisgeropoulos, Michael W. Ratzloff, Ekaterina M. Stroeva-Dahl, Sarah Hasan, Febin Varghese, Jacob H. Artz, John W. Peters, David W. Mulder, Paul W. King

**Affiliations:** † Biosciences Center, 53405 National Renewable Energy Laboratory, Golden, Colorado 80401, United States; ‡ Institute of Biological Chemistry, 187873 Washington State University, Pullman, Washington 99163, United States; § Department of Chemistry and Biochemistry, University of Oklahoma, Norman, Oklahoma 73019, United States; ∥ Renewable and Sustainable Energy Institute (RASEI), University of Colorado Boulder, Boulder, Colorado 80303, United States

## Abstract

[FeFe]-Hydrogenases are structurally diverse enzymes that catalyze reversible H_2_ activation at a catalytic cofactor or H-cluster. The H-cluster is a [4Fe-4S] cubane linked by a cysteine thiolate to a diiron subsite containing unique CO, CN^-^, and dithiomethylamine ligands. The established H-cluster resting state of [4Fe-4S]^2+^-[Fe^II^-Fe^I^], or H_ox_, functions in H_2_ binding and oxidation, or by proton-coupled reduction initiates H_2_ evolution. In contrast, in Clostridium pasteurianum [FeFe]-hydrogenase III (CpIII) the resting state of the H-cluster is fully oxidized, [4Fe-4S]^2+^-[Fe^II^-Fe^II^], or H_ox+1_. To begin to understand if H_ox+1_ has a role in the mechanism of CpIII, we determined the spectroscopic and redox properties of CpIII H-cluster states under catalytic conditions. CpIII poised in H_ox+1_ and either equilibrated under 1 atm of H_2_ or reduced with sodium dithionite, resulted in a mixture of reduced states including H_ox_ (*E*_m_^8^ = −407 mV), H_trans_-like [4Fe-4S]^+^-[Fe^II^-Fe^II^] (*E*_m_^8^ = −418 mV), H_red_ [4Fe-4S]^+^-[Fe^II^-Fe^I^], and H_redH+_ [4Fe-4S]^2+^-[Fe^I^-Fe^I^] (*E*_m_^8^ = −455–480 mV). Under H_2_ the population of the H_trans_-like state was >20-fold higher than H_ox_, implicating a role in CpIII catalysis. Unlike other enzymes, there was no spectral evidence of fully reduced states, such as H_sredH+_ ([4Fe-4S]^+^-[Fe^I^-Fe^I^]) or H_hyd_ ([4Fe-4S]^+^-[Fe^II‑^Fe^II^]-H^–^). Thus, while the H-cluster states of CpIII encompass most of the catalytic intermediates, it is either unable to form H_sredH+_ and H_hyd_, or these states are highly destabilized in CpIII. Thus, these results demonstrate that catalytic intermediates of reduced CpIII differ from the typical intermediates of other catalytic [FeFe]-hydrogenases and may explain the catalytic preference for H_2_ production.

## Graphical abstract

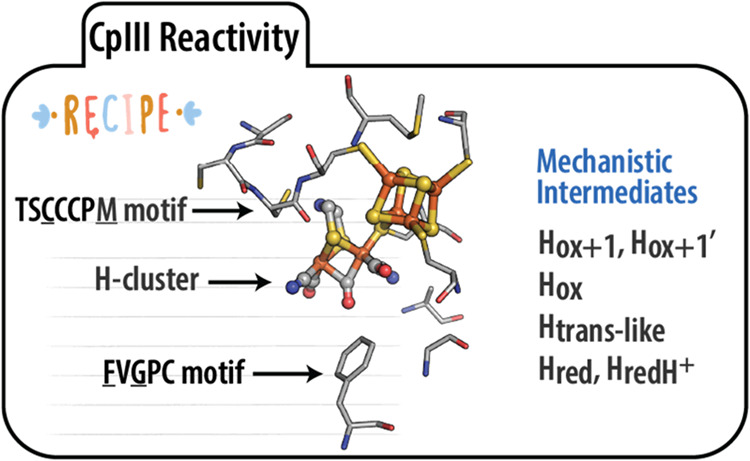

## Introduction

Hydrogenases are enzymes that catalyze reversible H_2_ activation—coupled to the oxidation–reduction of electron carrier molecules—to function in energy metabolism and microbial pathogenicity.^1^ The [FeFe]-hydrogenases are distinguished by having a conserved, organometallic cofactor, termed the H-cluster, which functions as the site of H_2_ activation.^2^ The H-cluster is composed of a [4Fe-4S] subsite linked by a cysteine thiolate to a diiron [2Fe] subsite.^3,4^ Defining features of the diiron subsite are CO and CN ligands that stabilize each Fe as low-spin Fe^I^/Fe^II^ and a bridging dithiol-dimethylamine ligand that functions in proton transfer.^5^ In addition to the H-cluster, many [FeFe]-hydrogenases contain diverse complements of accessory iron–sulfur clusters (F-clusters) that mediate the exchange of electrons with external redox partners.

The range of reactivities observed among different classes of [FeFe]-hydrogenases have been hypothesized to result from variations possibly in the secondary coordination environment of the H-cluster (Figure 1),^6,7^ or in the nature of the F-clusters.^8–13^ Support for the role of the H-cluster environment comes from studies on a variety of engineered enzyme variants,^7,14–18^ one example beingClostridium pasteurianum [FeFe]-hydrogenase III (CpIII).^6,19,20^ Extensive sequence alignment studies of [FeFe]-hydrogenases have led to classification into four main phylogenetic groups, A–D.^2,14,21–26^ CpIII is classified as a Group B [FeFe]-hydrogenase, which differs from more well-studied Group A enzymes in the sequence composition of the H-domain that coordinates the H-cluster (Figure 1). For example, CpIII has a TSCCCP H-cluster binding motif versus a more standard TSCCP motif.^6,14,24^ The additional cysteine, C221, is located near the surface and C222, the proton-transfer cysteine, is located within hydrogen bonding distance to the H-cluster [2Fe] subsite (Figure 1).^27–29^ A CpIII variant with a deletion of C_223_, which in a CpIII AlphaFold model is expected to coordinate to the H-cluster [4Fe-4S] (Figure 1), resulted in a functional enzyme with attenuated electrochemical activity, demonstrating the importance of the additional cysteine residue to catalysis.^19^

**Figure 1:**
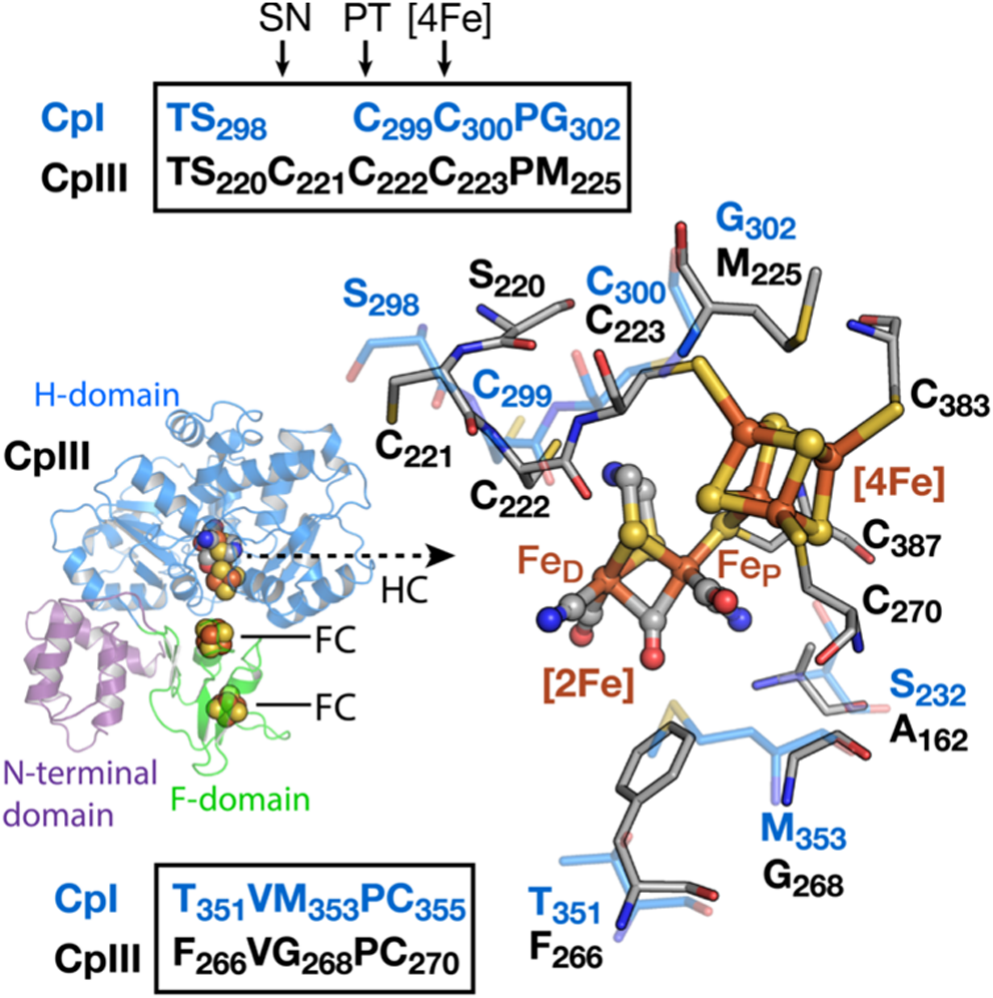
AlphaFold/AlphaFill structural model of CpIII and H-cluster binding region (black) superimposed onto the structure of CpI (blue, PDB ID 3C8Y). The CpIII F-domain (green) coordinates two accessory [4Fe-4S] clusters (FCs), and the H-domain (blue) coordinates the H-cluster (HC). H-cluster motif 1 (**top**) encodes the conserved Cys residues that function in coordination with the cubane subsite ([4Fe], C_223_) and proton-transfer (PT, C_222_). The model predicts that C_221_ is a supernumerary cysteine (SN). M_225_ of CpIII is predicted to occupy the same position as G_302_ of CpI to form a part of the electrostatic field near the [4Fe] subsite. Motif 2 (**bottom**) includes residues that form part of the electrostatic field of the diiron subsite ([2Fe]; F_D_, distal Fe of [2Fe]; Fe_P_, proximal Fe of [2Fe]). The F_266_/G_268_ pair of CpIII aligns with the T_351_/M_353_ pair of CpI, which results in an exchange of a polar CH_2_SCH_3_ side chain of M_353_ for a hydrophobic phenyl ring of F_266_ near the μ-CO of the diiron subsite of the H-cluster. The exchange of A_162_ of CpIII for S_232_ of CpI results in the loss of an H-bond interaction with the CN ligand proximal to the [4Fe] subsite in CpIII. Atomic coloring scheme: C, blue (CpI); C, gray (CpIII); N, blue; O, red; Fe, rust; S, yellow.

Along with the unique TSCCCP motif, variations in the residues of the other H-cluster motifs of CpIII further alter the outer coordination spheres of the two subsites (Figures 1 and S1). It has been hypothesized that these differences collectively alter the dielectric environment of the H-cluster in CpIII to impact stabilities of catalytic states.^6^ A striking example of this effect is that the resting state of CpIII is the most oxidized H-cluster state, [4Fe-4S]^2+^-[Fe^II^-Fe^II^] (or H_ox+1_).^6^ H_ox+1_ shares the same formal oxidation state as the H_inact_ state observed in Group A enzymes from *Desulfovibrio desulfuricans* (DdH) and Clostridium beijerinckii (CbA5H),^30–33^ and “State 1” that is observed in proton-transfer variants of the H_2_ sensing enzyme HydS from *Thermoanaerobacter mathranii* (*Tam*HydS).^34^ Both H_inact_ and State 1 are electron paramagnetic resonance spectroscopy (EPR) silent (*S* = 0) with H_inact_ assigned as a noncatalytic state. For DdH and CbA5H, the formation of H_inact_ is accompanied by reversible binding of an S-ligand at the [2Fe] subsite.^16,30,32,35–39^ It can also form in synthetically matured DdH by the irreversible binding of free CN^-^ that is a product of inefficient chemical reconstitution with artificial diiron compounds, or by the addition of a chemical CN^-^ donor.^40^

For CpIII, the H_ox+1_ state, is observed as the resting state of the recombinant, biosynthesized enzyme,^41–44^ isolated under anaerobic, reducing conditions,^6^ in the absence of sodium sulfide. Biosynthesis employs the natural maturase machinery for high-fidelity H-cluster synthesis, which unlike reported for chemical reconstitution,^40^ sequesters both CO and CN^-^ as protein-coordinated diiron precursors.^25,26,41,44–48^ Because H_ox+1_ is stabilized at potentials of ∼ −400 mV vs NHE) that are similar to the noncatalytic H_inact_ state of CbA5H (*E*_0_ = −383 mV vs NHE),^6,30^ raises the question of the role of H_ox+1_ in CpIII and the nature of the reduced states that comprise the catalytic cycle.

Herein, we tested this hypothesis of mechanistic plasticity by examining the reduced states of CpIII using electron EPR and Fourier transform infrared (FTIR) spectroscopy. Specifically, we examined the identity and reduction potentials of H-cluster intermediates in CpIII poised under catalytic conditions. We also tested whether the predicted proton transfer cysteine, C222, was involved in stabilizing the H_ox+1_ state, as demonstrated in CbA5H, by changing Cys to a Ser. The collective results demonstrate that CpIII stabilizes H-cluster oxidation states differently than the Group A [FeFe]-hydrogenases. How these differences may account for the unique reactivity of CpIII is discussed and signifies an underlying role of the coordination sphere in controlling the catalytic properties of the cofactor.

## Materials and Methods

### Enzyme Modeling

The AlphaFold 2 model of C. pasteurianum CpIII (A0A0H3J7C7)^49,50^ was used in AlphaFill^51^ at the 25% identity threshold to populate H- and F-cluster positions. The resulting structure contained multiple possibilities for F-cluster locations. Based on manual inspection of cluster placement chains, only chains U and N were retained as the F-clusters, as they best fit the position of the accessory iron–sulfur cluster sequence motifs in the F-domain of CpIII. Positioning of the diiron site of the H-cluster ([2Fe]_H_) was further based on superimposition of [2Fe]_H_ from the X-ray crystal structure of [FeFe]-hydrogenase CpI (PDB ID 3C8Y).^52^

### Enzyme Expression and Purification

CpIII (Genbank accession number, WP_003447632) was expressed and purified as previously described and stored anaerobically at 4 °C until usage.^6,20^ The CpIII C222S variant construct was generated from the pETDuet-1 CpIII plasmid by site-directed mutagenesis (GeneScript). Expressions and purifications were performed as described previously,^6^ and analyzed by SDS-PAGE. Concentrations were determined by Bradford assay. Specific activities of purified enzymes were assayed by H_2_ evolution from sodium dithionite-reduced methyl viologen (MV).^6^ The CpIII C222S variant displayed a ∼10-fold lower activity than native at 30 μmol H_2_ min^–1^ mg^–1^ compared to 305 ± 58 μmol H_2_ min^–1^ mg^–1^,^6^ consistent with the effect of the Cys-to-Ser exchange of proton-transfer residue in other [FeFe]-hydrogenases.^20^

### FTIR and EPR Sample Preparation

#### FTIR

Auto-oxidized (AO) CpIII (Figures 2, 3, 5, and S2) was prepared by G-25 buffer exchange into 50 mM Tris pH 8, 200 mM NaCl, and 5% glycerol and stored at 4 °C. Thionine oxidation of CpIII was carried out by treatment of the as-isolated enzyme by a 1 mM final concentration of thionine (Figure 5). Treatments with either 100% H_2_ or D_2_ for FTIR (Figure 3) were performed by headspace exchange using 10 vacuum/sparge cycles on a Schlenk line. AO CpIII C222S was prepared by storage of the as-isolated enzyme at 4 °C for a period of 11 days (Figures S7 and S8) or two months (Figure 5) without further reduction. Samples (15 μL) were loaded onto CaF_2_ windows with 15 μm spacers, and spectra were collected in a custom-built, airtight sample holder.

**Figure 2:**
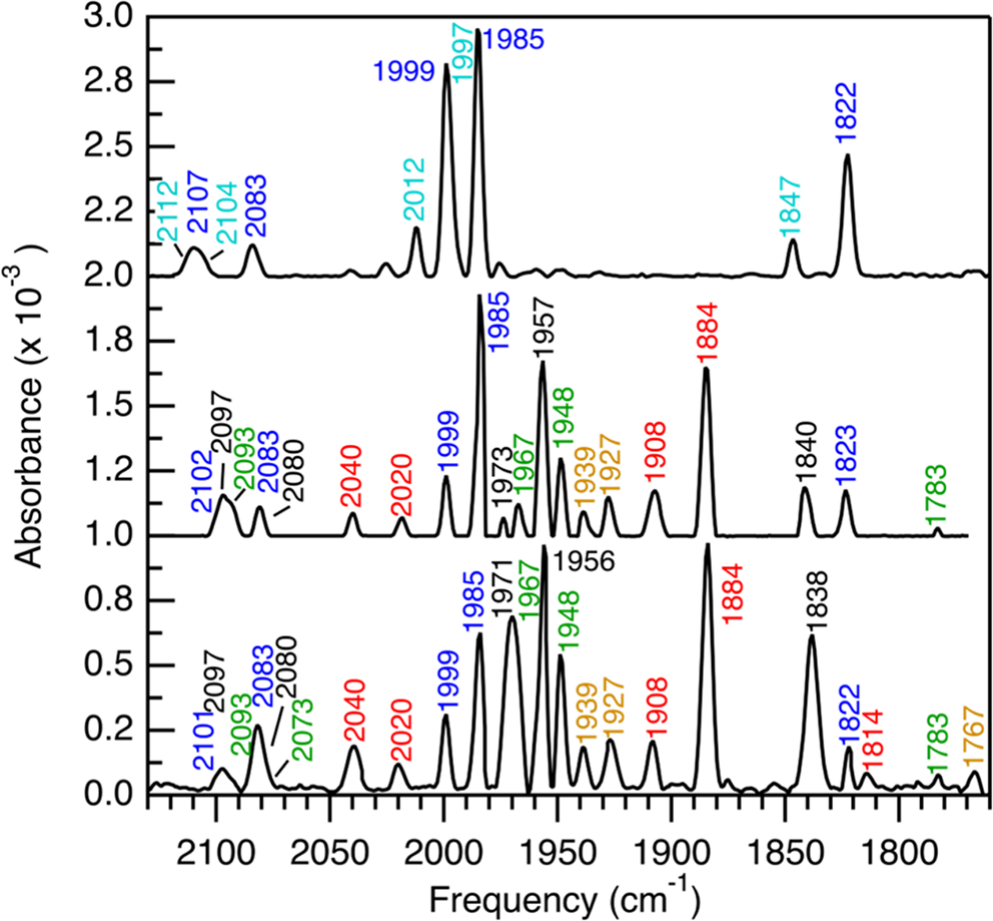
IR spectra of oxidized and H_2_-treated CpIII. Top, spectrum of auto-oxidized (AO) CpIII (100 mg mL^–1^) collected at 298 K. **Middle** and b**ottom**, AO sample treated under 1 atm of 100% H_2_, pH 8, and collected at 195 K (**Middle**) or 10 K (**Bottom**). The signals are H_ox+1_′ (cyan), H_ox+1_ (blue), H_ox_ (green), H_trans_-like (black), H_red_ (orange), and H_redH+_ (red).

**Figure 3:**
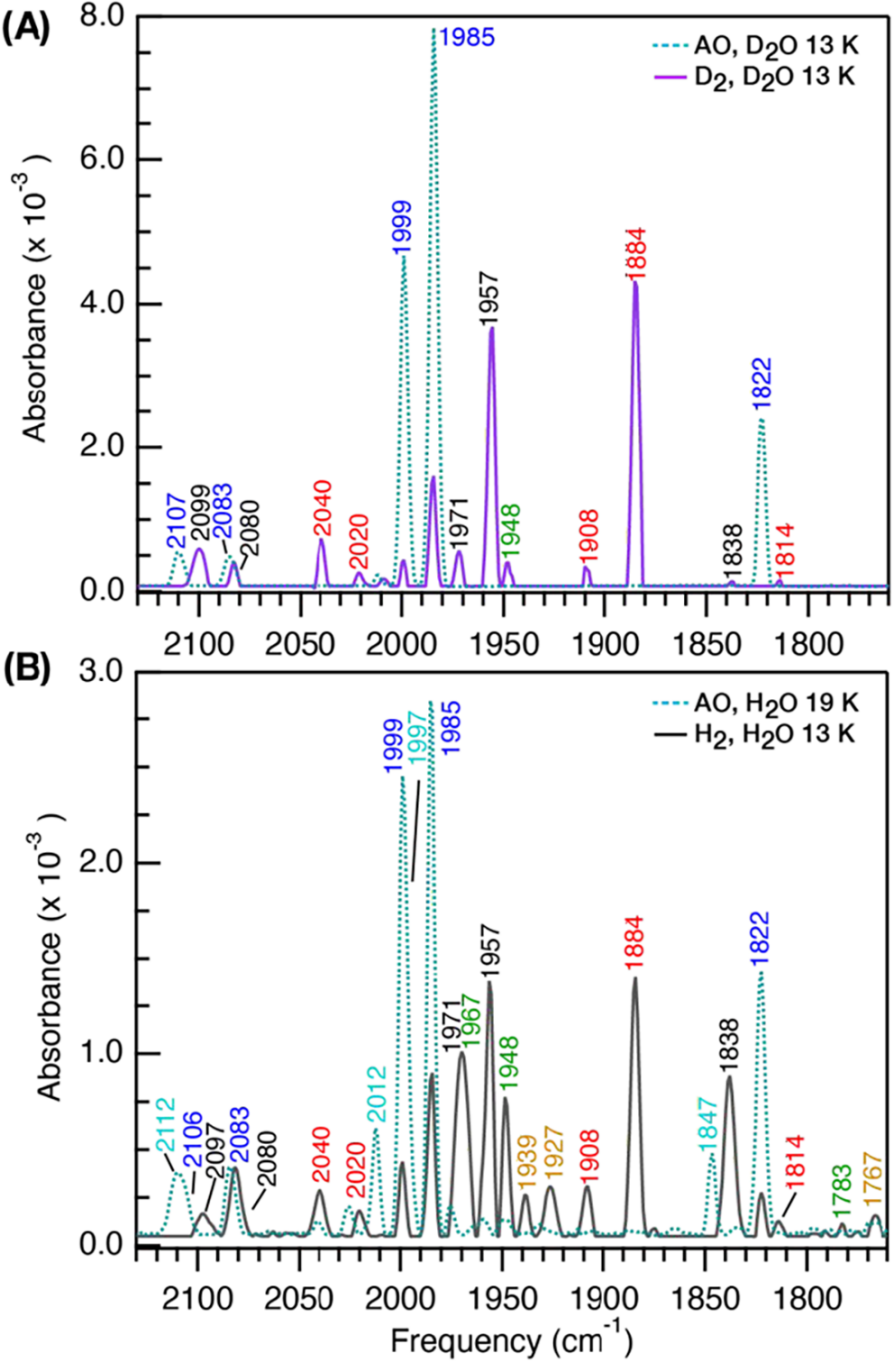
FTIR spectra of CpIII treated under 100% D_2_ or 100% H_2_. AO CpIII (100 mg mL^–1^) was prepared in D_2_O buffer, pD 8 (**A, aqua**), or H_2_O buffer, pH 8 (**B, aqua**). Treatment on a Schlenk line under 1 atm of 100% D_2_ (**A, purple; 4x intensity**) or 100% H_2_ (**B, black**). H-cluster oxidation states; H_ox+1_ (blue), H_ox+1_′ (cyan), H_ox_ (green), H_trans_-like (black), H_red_ (orange), and H_redH+_ (red). Spectra were collected at 298 or 13 K.

### EPR

AO CpIII and CpIII C222S were prepared as described above (Figures S3 and S8). CpIII treated with H_2_, and sodium dithionite (Figures 4 and S3) was prepared by exchanging the AO enzyme into buffer containing 10 mM sodium dithionite and then subjected to headspace exchange into 100% H_2_ as described above. Reduction with H_2_ alone (Figure 4, middle) was performed by headspace exchange of AO CpIII with 100% H_2_ using 10 vacuum/sparge cycles on a Schlenk line and incubated overnight at 4 °C prior to freezing. The CO-treated CpIII (Figure 4, bottom) was generated by first performing H_2_ reduction, incubated overnight, and the following day an aliquot placed in a septum-sealed conical vial (N_2_ headspace) and then sparged twice with 100% CO. 200 μL samples were loaded anaerobically into X-band EPR tubes of 3 mm ID (Wilmad lab glass, 707-SQ-250 mm). The preparation of CpIII redox titration samples (Figures S4–S6) included a cocktail of redox mediators as described.^6^ All potentials are reported in mV vs NHE.

**Figure 4:**
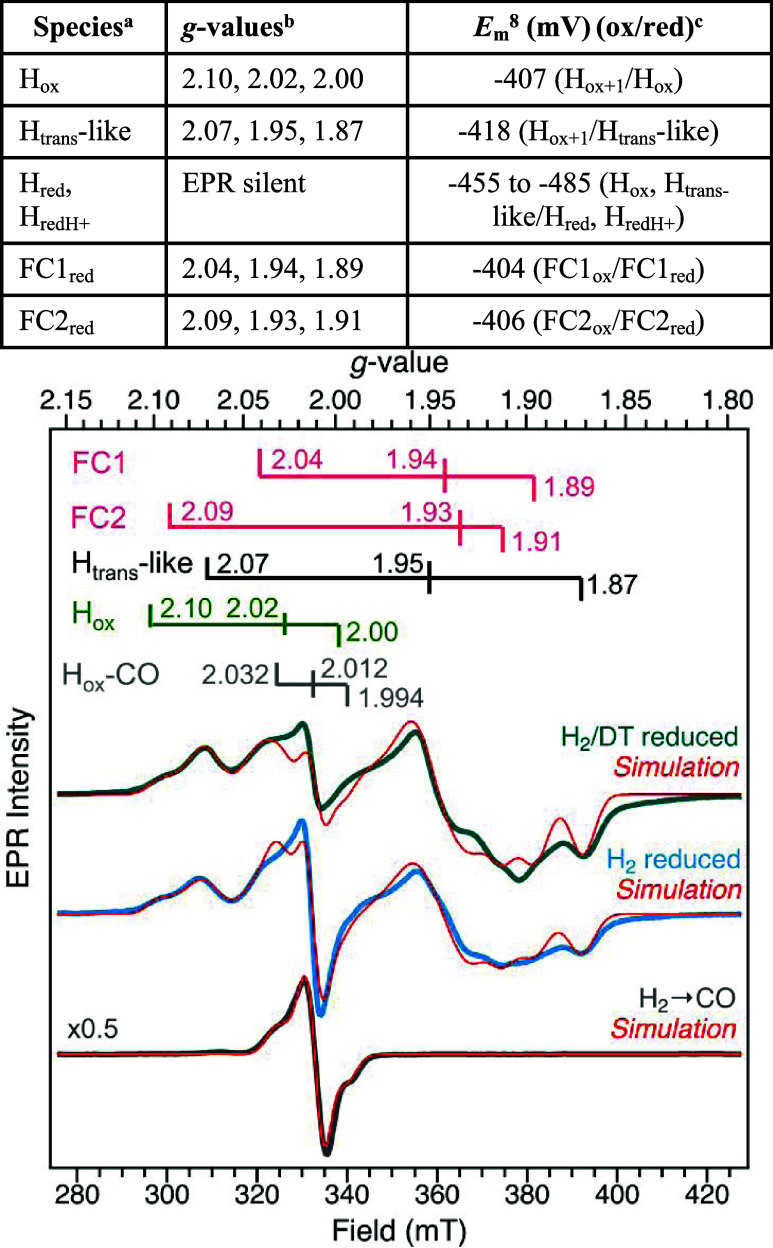
CW X-band EPR spectra of CpIII. AO CpIII was treated with either 1 atm of 100% H_2_ in the presence of sodium dithionite (*T* = 20 K, *P* = 1 mW), **Top trace**; 100% H_2_ and incubated overnight (*T* = 15 K, *P* = 1 mW), **Middle trace**; or 100% H_2_ (overnight) followed by sparging under 100% CO (*T* = 15 K, *P* = 1 mW; data shown at half-intensity for visualization). Spectral simulations (**red traces**) comprised of H-cluster and F-cluster signals were performed using parameters in Table S2, and *g*-values are shown at the top.

### FTIR and EPR Spectra Collection and Processing

#### FTIR

Spectroscopy was performed at 298 K (spectra collected with an MCT detector) or at cryogenic temperatures (ColdEdge Technologies cryostat; spectra collected with a high-D* MCT detector) on an N_2_-purged Nicolet 6700 Spectrometer (Thermo Fisher Scientific) as previously described.^53^ Final spectra consist of 512 coadded scans at 2 cm^–1^ resolution. Baselines were manually corrected using sequential spline and linear functions, and in some cases water vapor background subtraction, in either the OMNIC software (Thermo Fisher Scientific) or IgorPro v9.

#### EPR

X-band (∼9.38 GHz) CW EPR data were collected on an Elexsys E500 spectrometer equipped with a super high-Q resonator (Bruker), cryogen-free helium system (ColdEdge Technologies), and MercuryiTC temperature controller (Oxford Instruments, UK). Spectra were collected at various powers and temperatures (as indicated) by using a modulation frequency of 100 kHz and modulation amplitude of 10 G. EPR spectra were generally baseline corrected via subtraction of a polynomial function in IgorPro v9. For the full redox titration data set which is shown in reference 6 (data collected at *T* = 15 K with *P* = 1 mW) and used in conjunction with updated and new signal assignments to generate the current *E*_m_ analysis (Figure S4), the −29 mV spectrum was used to subtract a general buffer and redox-mediator background from all subsequent samples, with additional baseline correction performed as needed. We note this mediator background signal continued to grow at lower potentials and is accounted for in simulations of the 20 K data by the inclusion of an axial-type signal (see the Simulation of EPR Spectra section).

### EPR Signal Identification

To obtain temperature-normalized spectra for analysis, variable-temperature CW spectra shown in Figures S3 and S5 were corrected for the Curie Law through multiplication of the spectra signal intensity by their respective collection temperatures. A power of 1 mW was commonly used, as qualitative analysis indicated minimal power saturation of any species when using this power to collect in their respective *T*_opt_ ranges.

#### H_ox_-CO Signal

Initial attempts to simulate the CO-treated CpIII EPR signal (Figure 4) as a single rhombic-type species were not successful. The temperature-dependent behavior of the signal was then assessed which revealed differential temperature-dependent intensity changes for the central derivative feature compared to the wings of the spectrum. Further analysis provided evidence of a second unknown signal with distinct temperature-dependent behavior, which underlies the H_ox_-CO signal. Simulation of the data using both the rhombic H_ox_-CO signal (*g* = 2.0315, 2.012, and 1.9935) and a second signal (*g* = 2.017, 2.008, and 2.008) (Table S2) produced high-quality fits at all temperatures (see the Simulation of EPR Spectra section).

#### H_ox_ Signal

The H_ox_ signal of CpIII was first described in reference,^6^ which used simulation of a potentiometric sample poised at −399 mV (*T* = 15 K; *P* = 1 mW) to determine a H_ox_ *g*-tensor of *g* = 2.10, 2.04, 1.99. As the first turning point of the H_ox_ signal (*g*_1_ = 2.1) represents the low-field edge of the spectrum, it was used throughout as the primary feature to track H_ox_ in variable-temperature or potentiometric data. In this manner, the *T*_opt_ of the H_ox_ signal was determined from the H_2_ reduced data to be in the range of 40–50 K (Figure S3; Table S2). This temperature response was confirmed with analysis of the −399 mV sample variable-temperature spectra (Figure S5), which also displayed a high population of H_ox_. We also note the H_ox_ signal described in reference 6 is refined here as *g* = 2.10 2.024 1.998 (see the Simulation of EPR Spectra section).

#### H_trans_-like Signal

The H_trans_-like EPR signal was first identified from the H_2_ reduced data (Figures 4 and S3) using additional features at *g* ∼ 2.07 and *g* ∼ 1.87 (modeled as the *g*_1_ and *g*_3_ values of a rhombic signal, respectively). Similar to the approach used to analyze H_ox_, the H_trans_-like feature at *g* ∼ 1.87 was used primarily for analysis due to its position outside the other spectral features. Since signals from the F-clusters and H_ox_ considerably overlap the H_trans_-like feature at *g* ∼ 2.07, this feature was examined only as a comparison to aid in analysis. In this way, a *T*_opt_ of 20 K was determined for H_trans_-like, with agreement from monitoring both features in the H_2_ reduced data. Analysis of the H_trans_-like signal in the −442 mV variable-temperature data set confirmed a *T*_opt_ = 20 K, with the features at *g*_1_ and *g*_3_ displaying *T*_opt_ ranges of 15–20 K and 20–30 K, respectively. The temperature range on analysis from −442 mV data is not surprising given the lower simulated population of H_trans_-like signal in the −442 mV spectrum at 20 K compared with H_2_ reduced (*vide infra*, Table S3).

#### F-Cluster Signals

Analysis of the spectra presented in this work allowed for further deconvolution of the F-cluster features (previously assigned)^6^ into two discrete sets of signals, FC1 and FC2 (Table S2). These signals were most clearly resolved in the −399 mV spectra (Figures S5 and S6), particularly around their *g*_3_ values (simulated as FC1 = 1.895; FC2 = 1.914). For more reduced samples (i.e., −442 mV, H_2_-treated), the additional intensity was present in this region of the spectrum, as well as surrounding the *g*_2_ of FC1 (*g* ∼ 2.04), which hindered the resolution of the signals (Table S2, Figures S5–S6). Therefore, the temperature-dependent behavior of the F-cluster signals was analyzed using primarily the −399 mV spectra at the specific *g*_3_ values given above. In these data, we note an apparent slight shift of the FC2 feature (*g*_3_ = 1.914) to a higher *g*-value when going from 20 to 30 K (Figure S5). This could be due to additional spectral effects from spin–spin coupling between the FC1 and FC2 clusters, which generally resolve better with colder temperatures. This explanation is corroborated by analysis of the H_2_ reduced and −442 mV spectra (Figures S3 and S5). While these signals display far less resolution in the spectral region due to the increased signal intensity, they indicate a similar temperature-dependent shift of the *g*_3_ feature. Given these observations and the complexity of deconvoluting possible additional spin–spin coupling spectral effects, no difference was rigorously determined in the temperature-dependent behavior of the CpIII F-cluster signals obtained from these spectra, with a *T*_opt_ range of 20–30 K assigned for both FC1 and FC2 (Table S2). Likewise, we were unable to resolve any significant difference in *E*_m_ values (Table 2; Figure S4) for the two cluster signals (*vide infra*).

### Simulation of EPR Spectra

Simulations were performed using the EasySpin toolbox and its core function “pepper” within Matlab version R2020A (MathWorks) with full simulation parameters given in Table S2.^54^ A gStrain parameter was used to replicate line broadenings. Spectra were not normalized prior to the simulation. Table S3 gives the relative populations of each EPR species, obtained from fitting the simulated intensity of each signal to the experimental spectral intensity, with the contribution of each species reported as a % of the total simulated intensity.

Using the *T*_opt_ values determined from analysis of temperature-normalized data (Table S2), a maximal intensity is obtained for the H_trans_-like and FC signals between 20 and 30 K, and therefore data collected at 20 K were chosen for simulation to further deconvolute signal assignments and determine relative spectral contributions. Although the H_ox_ signal (*T*_opt_ = 40–50 K) experiences saturation effects at this temperature, the use of only 20 K data allows for a comparison of relative changes to the simulated H_ox_ contribution across the data set.

For simulation of the CO-treated data at 15 K (Figure 4) the H_ox_-CO signal accounted for ∼ 75% of the total simulated spectrum with the underlying signal accounting for the remaining 25%. This ratio of 3:1 was similar to the ratio from the simulation of the H_2_/sodium dithionite reduced spectrum collected at 20 K (Figure 4), however in this case we note the H_ox_-CO and underlying signal combined account for only ∼2% of the total simulated spectral weight (Table S3). Simulations of redox titration data at 20 K (Figure S6) were performed with the inclusion of an axial-type radical signal with *g*_⊥_ = 2.018 and *g*_||_ ∼ 2.01 (Table S2) to account for the contribution of redox mediators. This same background signal accounted for ∼2.4% (−399 mV), ∼1.8% (−442 mV), or ∼10% (−351 mV) of the total simulated intensity for the spectrum. We note the overall simulated signal intensity for the −351 mV spectrum is low compared to what is simulated for other samples (Table S3), which is unsurprising given the low overall signal intensity and resolution of H-cluster and F-cluster signals at this potential (Figure S5 and S6).

During these analyses, we also noted a clear difference in the quality of fit between the simulation of the less reduced (i.e., −399 mV) and the more reduced (i.e., −442 mV, H_2_-treated) data. This was particularly true in the region of *g* ∼ 2.02, where multiple species overlap, and at *g* ∼ 1.9, where both FC1 and FC2 contribute. Spin–spin coupling between reduced FC1 and FC2 (*vide supra*) would contribute additional spectral complexity not accounted for by our simulations and would also be expected to impact the quality of the spectral fit more as reduction continues. We also note that the H_ox_ signal contribution in the middle of the spectrum (*g*_2_ = 2.024) might impact the quality of the fit in this region due to the saturation effects, although the absence of any H_ox_ signals near *g* = 1.9 precludes this as the sole explanation for the additional experimental intensity.

The exact *g*-values used to simulate each spectrum are given in Table S2. Slight variations on the order of Δ*g* ≤ 0.002 were present in some of the *g*-values simulated in different samples. These shifts can generally be explained by the complexity of the spectral region due to overlapping species or other physical reasons, such as contributions from spin–spin coupling. As both F-clusters are reduced, spin–spin coupling between FC1 and FC2 can influence the line-shape and even position of the F-cluster spectral features. The exact measure of the spectral effect is dependent on the strength of coupling and the degree of reduction of both clusters, and effects can also be anisotropic (influencing parts of the spectrum differently) depending on the geometry of the magnetic interaction. As H_ox_ was simulated under conditions of temperature-saturation, a combination of fitting and global analysis that included higher temperature data was used to first refine the published *g*-tensor from Artz et al.^6^ and the resulting *g*-values reported in Table S2 were used in subsequent simulations without additional fitting. The *g*_1_ feature of the H_trans_-like signal displayed the only shift (Δ*g* = 0.0025) outside the range reported above, although this is in essence a difference between the H_2_ reduced (*g*_1_ = 2.072) and potentiometric (*g*_1_ ∼ 2.07) data. These differences could result from the different methods of reduction and/or greater population of H_trans_-like in the H_2_ reduced sample. The derivative feature of the rhombic H_trans_-like signal (i.e., *g*_2_) was not fully resolved in any of the spectra analyzed here, however, comparison across data sets and reduction treatments showed that additional intensity was present at *g* ∼ 1.95 which correlated with the other H_trans_-like features. The poor resolution of the feature made exact assignment difficult, and therefore, the *g*-value was first allowed to fit generally in this region. From these results, the value of *g*_2_ = 1.952 (Table S2) was determined for use in subsequent simulations that reproduced the shape of the region relatively well (Figures 4 and S6).

Given the multiple, overlapping components in these data and the lack of additional constraints on fitting parameters to resolve the shifts described above, we report the final assigned signals of each species (except H_ox_-CO) with *g*-values rounded to the nearest hundredth in the callouts at the top of Figures 4, S3, and S5–S6. The H_ox_-CO signal was obtained from fitting of the data using only two components that resulted in high-quality simulations and is reported in the third decimal place. For simulation of the CpIII C222S variant in Figure S8 we include an additional resolution for *g*-values when simulated approximately halfway between the hundredth values.

### *E*_m_ Analysis from 15 K EPR Titrations

Potentiometric titration of CpIII poised from −29 mV to −442 mV previously determined the CpIII H_ox_ signal to have an *E*_m_^8^ = −389 mV vs NHE.^6^ A sample poised at −460 mV was also prepared and included in the *E*_m_ analysis of this work (Figure S4).

Potential-dependent changes in H-cluster or F-cluster signal intensities were monitored using the same features as those for temperature-dependent analyses (H_ox_, *g* = 2.1; H_trans_-like, *g* = 2.07; FC1, *g* = 1.895; FC2, *g* = 1.914). The *n* = 1 Nernstian behavior of the resultant data was then assessed by fitting the data to either eq 1 (FC1 and FC2) or eq 2 (H_ox_ and H_trans_-like);

1 $$f\left(x\right)=y_{0}+\frac{\max imum\;EPR\;signal}{1+e^{\left(x-E_{m}\right)F/RT}}$$

2 $$f\left(x\right)=y_{0}+\frac{\max imum\;EPR\;signal}{1+e^{\left(x-E_{m}1\right)F/RT}+e^{\left(E_{m}2-x\right)F/RT}}$$

using a custom fit function in IgorPro v9 (*y*_0_ = *y*-axis offset; *x* = potential in mV; *E*_m_ = midpoint potential in mV; F = Faraday constant = 96,480 C·mol^–1^; R = Gas constant 8.314 J·K^–1^·mol^–1^; T = Temperature in K).^55^ The 95% confidence band of each fit (where model points are expected to fall with a 95% or greater probability) is included (Figure S4). The H-cluster signal of H_ox_ appeared not to reach a maximum intensity or level off before decreasing, indicating an equilibrium process due to steady-state conditions at the lowest potentials. Likewise, the H_trans_-like signal displayed minimal intensity change at the end of the titration, although a significant decrease as observed for H_ox_ was not apparent, in line with a slightly lower *E*_m_ of formation for H_trans_-like. Therefore, these data were fit to eq 2, which includes an expression for a second Nernstian process representing the H_ox_, H_trans_-like/H_red_, and H_redH+_ couple. We note the lack of data constraining the fit of this second redox couple and present the results only to provide a general range.

### Population Analyses from Spectral Simulations

The results of simulating select potentiometric data (Figure S6) are shown in Tables S2 and S3. Given the low signal quality in the −351 mV data, we refrain from ascribing too much significance to the simulated weights at this potential. Instead, we have included these data to illustrate the lack of any discernible H_trans_-like contribution and provide context for the subsequent population increases simulated for all species.

Given its higher *T*_opt_, it is likely the amount of H_ox_ is slightly underrepresented in the simulated weights (Table S3), however, examination of the 40 and 50 K data in the temperature-dependent data set of Figure S5 corroborate the trend of H_ox_ contribution from 20 K simulations. While the 20 K simulations show a similar simulated weight of H_ox_ for −399 and −442 mV samples (Table S3), the actual percent of the total simulated weight decreases from −399 to −442 mV as spectral intensity from other signals increases. The simulated weight of H_ox_ at −442 mV is also complicated by additional spectral intensity contributions from possible F-cluster spin–spin coupling at this potential. These results are in line with the *E*_m_ analysis, which indicates a decrease in the H_ox_ population at lower potentials that can be described with an *E*_m_ ∼ −455 mV (Figure S4). Also, in agreement with the *E*_m_ analysis, the H_trans_-like population determined from the simulated weights appears to increase as potential is lowered, in both absolute simulated intensity and as a percent contribution to the overall simulated intensity. The F-cluster trends are less straightforward, in a manner that matches the observed complexities and likely spectral contributions from spin–spin coupling.

## Results

### IR Spectral Properties of Reduced CpIII

During a catalytic cycle, the H-cluster cycles through reduced and oxidized intermediates, which can be detected by Fourier transform infrared (FTIR, or IR) and electron paramagnetic resonance (EPR) spectroscopies. In IR spectroscopy, the reduction–oxidation of the H-cluster subsites can be detected as changes in *v*CO and *v*CN band frequencies. The IR and EPR spectra also can enable the resolution of isoelectronic states that differ in the distribution of electronic spin across the cluster, like H_ox_ and H_trans_ ^56^ or H_hyd_ ^42,43,57,58^ and H_sredH+_,^59–62^ because the H-cluster EPR signals and diiron subsite *v*CO and *v*CN band frequencies are highly sensitive to the base electronic structure. IR is also powerful for detecting otherwise EPR silent, diamagnetic states, for example, the fully oxidized H-cluster^33^ and the one-electron reduced H_red_ (defined here as [4Fe-4S]^+^-[Fe^II^-Fe^I^]) or H_redH+_ (defined here as [4Fe-4S]^2+^-[Fe^I^-Fe^I^] states.^62–65^

To begin assigning the reduced H-cluster states of CpIII, the IR spectrum of the anaerobically oxidized (auto-oxidized, AO) was collected. The AO spectrum primarily consisted of the H_ox+1_ state, [4Fe-4S]^2+^-(Fe^II^-Fe^II^),^6^ composed of a distinct set of *v*CN bands at 2106 and 2083 cm^–1^, *v*CO bands from terminally bound CO ligands (*t*-CO) at 1999, 1985, and the *v*CO band of a bridging CO ligand (μ-CO) at 1826 cm^–1^ (Figure 2 and Table 1). In some oxidized samples, a second set of *v*CO bands was observed at 2012, 1997, and 1850 cm^–1^, which was assigned to a second H_ox+1_ state (Figures 2, 5, and S2) referred to here as H_ox+1_′ (Table 1).

**Table 1:** IR Signals of the CpIII H-Cluster Oxidation States

**H-cluster**	***t*** **-CN** ^a^	***t*** **-CO** ^a^	***μ*** **-CO** ^a^
H_ox+1_′	2111, 2104	2012, 1997	1850
H_ox+1_	2107, 2083	1999, 1985	1826
H_ox_	2093, 2073	1967, 1948	1783
H_trans_-like	2097, 2080	1971 1956	1838
H_red_	2092, 2072	1939, 1927	1767
H_redH+_	2040, 2020	1908, 1884	1814
H_ox‑CO_	2103, 2090	2009, 1978, 1959	1795

a Assignments for H_ox+1_ and H_ox_, 298 K spectra in Figure 2; H_trans_-like, H_red_, H_redH+_, 13 K spectra Figure 3; H_ox_-CO, Figure S2.

**Figure 5:**
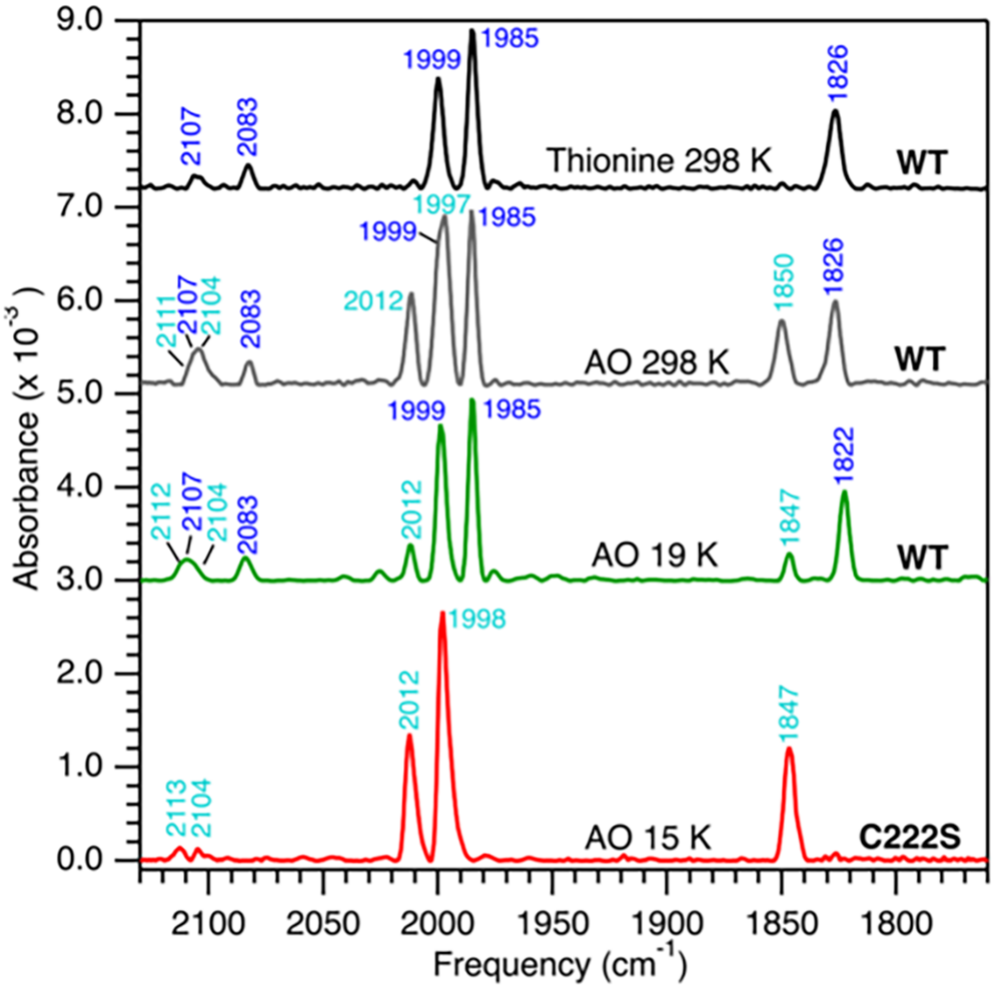
FTIR spectra of oxidized CpIII and CpIII C222S variants. Thionine oxidized CpIII collected at 298 K (**black**); AO collected at 298 K (**gray**) or 19 K (green); AO CpIII C222S collected at 13 K (**red**). The *v*CO and *v*CN modes of H-cluster states are labeled as H_ox+1_ (blue) and H_ox+1_′ (cyan). Samples were anaerobically purified in buffer with sodium dithionite (2 mM, pH 8), allowed to oxidize at 4 °C over a period of days, and exchanged into dithionite-free buffer prior to spectrum collection. WT = wild-type.

Equilibration of AO CpIII under 1 atm of H_2_ resulted in significant changes in the *v*CO and *v*CN band frequencies, consistent with the reduction of the AO sample into a mixture of reduced states with different electronic structures (Figures 2 and S2). The use of cryogenic temperatures had two effects; (i) it reduced the rate of turnover, which led predominantly to the H_ox+1_ state in room temperature spectra, and (ii) led to less line broadening of the *v*CO bands for improved resolution of spectral signals. It also revealed a temperature sensitivity of the μ-CO band frequencies of H_ox+1_ (Table S1), which we previously observed in [FeFe]-hydrogenase I of *Clostridium acetobutylicum* (CaI),^60^ and has been observed by others.^66^ For example, the comparison of spectra collected at 10 and 195 K led to resolution of spectral assignments to specific H-cluster oxidation states that were challenging to identify at 298 K (Figures 2, S2, and Table 1). In addition to H_ox+1_, there were signals consistent with the H_ox_, H_red_, and H_redH+_ states that are observed in other [FeFe]-hydrogenases. There was no detectable IR signal, indicative of a fully reduced H_sredH+_ state.

In addition to the H-cluster states identified above, there was an additional signal with a set of terminal *v*CO bands at 1971, 1956, and a μ-CO band at 1838 cm^–1^ (black labels, Figure 2). These bands are similar to signals assigned to either the hydride-bound state, H_hyd_ ([4Fe-4S]^+‑^[Fe^II^ Fe^II^]-H^–^), or an H_trans_-like state ([4Fe-4S]^+^-[Fe^II^ Fe^II^]), that have been observed in the Group A enzymes like CaI, CrHydA1 and DdH.^33,42,43,57,60^ Although these two states share the same base redox level, the H_hyd_ state contains a terminally bound hydride that is distinguishable by a frequency shift in the μ-CO band under H/D isotope editing.^29,42,57,60^ The shift is due to the *trans*-influence of a terminally bound hydride on the distal Fe atom (Fe_D_), which modulates the Fe_D_ π-backbonding with the μ-CO. We used this property to test for H_hyd_ by monitoring the 1838 cm^–1^ μ-CO peak under H/D isotope enrichment, looking for an isotope-dependent shift that would indicate this signal is from the H_hyd_ (rather than H_trans_) state.^42,67^ Equilibration of CpIII in the H_ox+1_ state under 1 atm of H_2_ (H_2_O) or D_2_ (D_2_O) (Figure 3) led to mixtures of oxidized and reduced states, including the 1971, 1957, and 1838 cm^–1^ signal. Although the levels of H_ox_, H_red_, and H_redH+_ enrichment differed, and there are isotope effects on *v*CO peak intensities, there was no evidence for an H/D isotopic shift in the μ-CO band frequency. Taken together, the results support the assignment of the 1971, 1956, and 1838 cm^–1^ signals in the 10 K spectrum (the μ-CO band at 1838 cm^–1^ shifts to 1842 cm^–1^ at 195 K) to a H_trans_-like state.

CO is known to react with [FeFe]-hydrogenases by binding to the H-cluster distal Fe site to form an oxidized CO-inhibited state, H_ox_-CO. We tested the reactivity of CpIII to CO by incubating either the AO or H_2_-treated CpIII with 100% CO and measuring the IR spectra. Whereas the AO sample was unreactive to CO (data not shown), when an H_2_ reduced sample, composed of a mix of H_ox+1_, H_trans‑_like, H_ox_ and H_red_ states, was incubated with CO it led to the conversion of the data to IR signal(s) consistent with the formation of H_ox_-CO (Figure S2). Thus, in contrast to the H_ox+1_ state in the AO sample, the H_ox+1_ state present in the H_2_ reduced sample reacts with CO.

### EPR Analysis of the H_ox_, H_trans_-like States, and Reduced F-Clusters

To further aid in identifying the distribution and nature of the reduced intermediates, EPR spectra of reduced CpIII were collected. Samples reduced by H_2_ either in the presence or absence of sodium dithionite (DT) showed approximately the same levels of the same signals (Figure 4, H_2_/DT vs H_2_) confirmed by simulation of the data (Tables S2 and S3). A slightly lower contribution from the H_ox_-CO species (1.7 vs 6.7%, Table S3) was observed in the H_2_/DT sample. Therefore, to simplify signal deconvolution, the EPR spectrum of H_2_/DT reduced CpIII was further analyzed.

In accordance with the IR spectra of CpIII in Figures 2 and 3, simulation and analysis of the EPR spectra collected at different temperatures (Figures 4 and S3) identified discrete H-cluster signals. Temperature optima (*T*_opt_) was used to make assignments of H_ox_, an H_trans_-like states. This also enables delineation of H_trans_,^56^ and reduced FC1 and FC2 F-cluster signals, that have similar rhombic line-shape (Tables 2 and S1, S2). A small population of a signal with a more axial line-shape consistent with a CO-inhibited state (H_ox_-CO) was also observed. This signal matched the signal at *g* = 2.032, 2.012, 1.994 of CpIII treated with H_2_ followed by 100% CO (Figure 4 and Tables S1, S2). Additional intensity in the H_2_ reduced data that was not accounted for by the simulation, particularly at *g* ∼ 1.9, can be attributed to spin–spin coupling between reduced FC1 and FC2 clusters (see Materials and Methods). Spin–spin coupling between clusters, which has been observed for other [FeFe]-hydrogenases, is expected due to the short distance (11 Å) and side-chain packing between the F-clusters, as predicted from the AlphaFold model (Figure 1).^9,20,68^ Consistent with the IR spectra results of reduced CpIII, there was no discernible H_hyd_ or H_sredH+_ like signal in these EPR spectra of reduced CpIII.

**Table 2:** CpIII H-Cluster and F-Cluster *E*_m_ Values^a^

**species** ^a^	***g*** **-values** ^b^	***E***_**m**_^**8**^ **(mV) (ox/red)** ^c^
H_ox_	2.10, 2.02, 2.00	–407 (H_ox+1_/H_ox_)
H_trans_-like	2.07, 1.95, 1.87	–418 (H_ox+1_/H_trans_-like)
H_red_, H_redH+_	EPR silent	–455 to −485 (H_ox_, H_trans‑_like/H_red_, H_redH+_)
FC1_red_	2.04, 1.94, 1.89	–404 (FC1_ox_/FC1_red_)
FC2_red_	2.09, 1.93, 1.91	–406 (FC2_ox_/FC2_red_)

a The species in the titration. The specific identity of H_ox+1_ or H_ox+1_′ species was not determined and noted here as H_ox+1_.

b Values from simulation of EPR spectra (*n* = 1) in Figures 4 and S6. Full parameters are listed in Table S2.

c The oxidized (ox) and reduced (red) species in the titration and their redox couple *E*_m_ values (mV vs NHE) at pH 8, obtained from the reanalysis of potentiometric EPR data reported in Artz et al.^6^ to generate Nernst curves (Figure S4) for all the resolved EPR signals in Figures 4, S3, and S5.

The FTIR and EPR spectra of H_2_ treated CpIII demonstrate that the H_trans_-like state and H_ox_ are in equilibrium under reducing conditions. Based on the evidence of a H_trans_-like state in CpIII, we re-evaluated the potentiometric EPR titration spectra reported in Artz et al.^6^ using the EPR signals for the H_trans_-like state and the FC1 and FC2 F-clusters resolved in this work (Figures S4–S6).

Fitting the intensity of the H_trans_-like signal (monitored at *g* = 1.872 as a function of potential), to the *n* = 1 Nernst equation (eq S1) gave an estimated *E*_m_^8^ = −418 mV (Table 2). This transition is nearly isopotential with the *E*_m_ of H_ox+1_/H_ox_, which was refined here as *E*_m_^8^ = −407 mV. The *E*_m_ values for the F-cluster signals were determined to be *E*_m_^8^ = −404 mV for FC1 and *E*_m_^8^ = −406 mV for FC2 (Table 2). Further analysis and simulations of spectra, including additional variable-temperature EPR data collected on select potentiometric samples (Figure S5), revealed the H_trans_-like state was stabilized to a higher level relative to H_ox_ in CpIII as potentials decreased to −442 mV (Figures S5 and S6), with a H_trans‑_like:H_ox_ ratio of 5:1 at −442 mV. This ratio increased when CpIII was equilibrated under H_2_ to a value of 28:1 (Table S3). Poising CpIII at potentials below −450 mV led to a decline in the H_ox_ signal while the intensity of the H_trans_-like signal appeared to level out (Figure S4). Fits of each of the potentiometric plots to a second *n* = 1 Nernstian process (eq S2) gave an estimated *E*_m_ range of −455 to −485 mV for reduction of H_ox_/H_trans_-like states to H_red_/H_redH+_ states. If the reduction of the H_red_/H_redH_ state of the H-cluster of CpIII behaves in a Nernstian manner, then the *E*_m_ for the formation of H_sredH+_ would be expected to be less than −485 mV.

### H_ox+1_ is Observed in a CpIII C222S Variant

As illustrated in Figures 1 and S1, the AlphaFold/AlphaFill structural model of CpIII predicts that each cysteine residue in the TSC_221_C_222_C_223_P motif has a specific function. C223 is predicted to be one of four cysteines that coordinate the [4Fe-4S]_H_ subsite, C222 to function in proton transfer with the [2Fe] subsite, and C221 to be a supernumerary cysteine distal to the H-cluster. The proximity of the proton-transfer cysteine to the H-cluster not only enables proton exchange but, in unique cases, can promote a direct interaction of the Cys-SH group with the [2Fe] subsite. This interaction is observed in the X-ray crystallographic structure of aerobically prepared [FeFe]-hydrogenase CbA5H of C. beijerinckii.^36^ Aerobically prepared CbA5H also stabilizes an inactive state, H_inact_, that is EPR silent and has a similar IR signal to the H_ox+1_ and H_ox+1_′ states of CpIII (Tables 1 and S3).^16,31,36^ Exchange of the conserved cysteine to either aspartic acid or alanine resulted in the loss of the ability to form the H_inact_ state and resistance to oxygen.^36^

We tested whether stabilization of H_ox+1_ in CpIII might be through a similar interaction of cysteine-SH with Fe_D_ by exchanging the predicted proton transfer cysteine, C222, to S222. A structurally conserved change that replaces the -SH group with an -OH group. The purified CpIII C222S variant had an H_2_ evolution activity of 30 μmol H_2_ min^–1^ mg^–1^, which was 10-fold lower than the reported 305 μmol H_2_ min^–1^ mg^–1^ of CpIII.^6^ FTIR of the AO CpIII C_222_S variant collected at 298 K (Figure S7) or 15 K (Figure 5) showed that the Cys-to-Ser change did not result in a loss of the H_ox+1_ states. Compared to AO CpIII, the FTIR spectrum of the AO CpIII C222S had a higher enrichment of the H_ox+1_′ state versus H_ox+1_. There were slight temperature-dependent shifts in the *μ-*CO band of H_ox+1_′ at 1847 (1850) and H_ox+1_ at 1822 (1826) cm^–1^ (Figures 5 and S7; frequencies from the 298 K spectrum of native CpIII are in parentheses). In addition, there were small contributions from H_ox_ and H_trans_-like H-cluster states observed in the 298 K spectrum (Figure S7). The corresponding EPR spectrum (Figure S8) showed the majority of reduced FC1 and FC2 signals with contribution from a similar composition of H-cluster states, consisting of H_ox_ and H_trans_-like signals (Figure S8, Tables S2 and S3).

## Discussion

Earlier EPR and IR experiments on CpIII had demonstrated that H_ox+1_ was stabilized at potentials below −418 mV based on the absence of H_ox_, which did not account for the possibility of a H_trans_ state as an intermediate. Here using EPR and IR to analyze reduced CpIII we were able to demonstrate that reduction of H_ox+1_ not only forms H_ox_ but also leads to an H_trans_-like state with an *E*_m_^8^ = −418 mV. Although the *E*_m_^8^ value is similar to H_ox_ (*E*_m_^8^ = −407 mV), at these potentials, the H_trans_-like state is more populated than H_ox_ (by as much as 28:1).^32,33^ By comparison, the H_trans_ state in DdH has a much more positive *E*_m_ than that of H_ox_, where H_ox_ is the dominant species at potentials near the value of the H^+^/H_2_ couple (*E*_m_ = −472 mV, pH 8, 1 atm H_2_).^33^ The stability of an H_trans_-like state in CpIII at a much lower potential of ∼ −418 mV implicates but does not prove that it may have a possible role in catalysis.

The most reduced H-cluster states in CpIII observed under the conditions of this study were H_red_ and H_redH+_ with an *E*_m_ range of −455 to −485 mV based on the modeled decay of H_trans_-like and H_ox_ EPR signals (Figure S4). Strikingly, there was no detectable EPR or IR signal of either a super-reduced, H_sredH+_ state, or a hydride-bound state, H_hyd_. While the lack of these states in this study does not necessarily rule out their occurrence, the distribution of reduced states thus far observed for CpIII is unlike most of the Group A, C, and D [FeFe]-hydrogenases. A comparison of the H-cluster intermediates of CpIII from these findings and comparison to those established for the Group A, C, and D enzymes is summarized in Table 3.

**Table 3:** Summary of the CpIII H-Cluster Redox States Compared to Group A, C, and D Enzymes

**state**	**group A, C, D**	**CpIII (Group B)**
oxidized^a^	H_inact_, [2^+^]-[II/II]	H_ox+1_, [2^+^]-[II/II]
	State 1, [2^+^]-[II/II]	H_ox+1_′, [2^+^]-[II/II]
	State 2, [2^+^]-[II/I]	H_ox_, [2^+^]-[II/I]
	H_air_-ox [2^+^]-[II/I]	
	H_ox_, [2^+^]-[II/I]	
	H_trans_, [1^+^]-[II/II]	H_trans_-like, [1^+^]-[II/II]
1 e^–^ reduced	H_red_, [1^+^]-[II/I]	H_red_, [1^+^]-[II/I]
	H_redH+_, [2^+^]-[I/I]	H_redH+_, [2^+^]-[I/I]
2 e^–^ reduced	H_sredH+_, [1^+^]-[I/I]	not detected
	H_hyd_, [1^+^]-[II/II]-H^–^	

a “Oxidized” here refers to species identified as inhibited or resting states, which for Group A, C, D enzymes includes H_inact_, H_air_-ox, H_ox_, and state 1 and state 2.^16,17,31,32,34^

The unique oxidation states of CpIII that have been established here coincide with several variations in H-cluster coordination sphere residues compared to those of Group A enzymes (Figure 1). This suggests a functional role for the coordination sphere in controlling the catalytic properties of the H-cluster. As shown in the AlphaFold/AlphaFill model of CpIII in Figure 1, there are specific structural differences in CpIII that include an F/G pair near the μ-CO of the [2Fe] subsite that replaces the T/M pair in CpI, where the methionine side chain is replaced by a more hydrophobic benzyl group of phenylalanine. Similar variations in these amino acid positions, either F/G or F/S, are observed in *Thermotoga maritima* HydS Group C bifurcating and *Tam*HydS Group D H_2_-sensing [FeFe]-hydrogenases, respectively.^15,69^ These enzymes exhibit decreased H_2_ evolution activities compared to Group A [FeFe]-hydrogenases as well as increased resistance to CO inhibition. *Tam*HydS variants that have changes in proton-transfer pathway residues stabilize an EPR silent State 1 that has an IR spectrum similar to H_ox+1_.^34^ CpIII and *Tam*HydS share similar μ-CO environments, suggesting there may be a connection between the local H-cluster environment and the ability to stabilize fully oxidized H-cluster states. Another example is the role of a methionine near the μ–CO in Group A enzymes that exist in different structural conformations. This flexibility is also observed as a change in the orientation of the Met side chain to μ-CO upon CO ligand binding at Fe_D_.^6,70^ Altogether, the composition and interaction of amino acids (i.e., Phe or Met) near the μ-CO ligand appear to be a mechanism by which [FeFe]-hydrogenases modulate the stabilization of H-cluster intermediates and catalytic reactivity.

Another unique feature of CpIII is the presence of a supernumerary cysteine, or C221, that is located within the TSCCCP motif and absent in Group A, C and D enzymes.^71–73^ A recent study tested the role of an extra cysteine in the reactivity of CpIII using electrochemical kinetics.^19^ The results demonstrated that two independent, inactive species, I_1_ and I_2_ were formed at high (*E* ^0^ = −139 mV) and low (*E* ^0^ = −384 mV) potentials, respectively. Based on the similarity of the *E* ^0^ value of I_2_ to the *E*_m_^8^ = −389 mV of the CpIII H_ox+1_,^6^ the authors proposed that I_2_ and H_ox+1_ were the same H-cluster state. A deletion of C223, predicted to coordinate the [4Fe-4S] subsite (Figure 1) was used to convert the CpIII TSCCCP motif into a canonical TSCCP motif and resulted in a variant that lost 97% of the native CpIII activity as well as the ability to form the low potential, I_2_, state.^19^ Based on these results, the authors proposed that C222 in native CpIII, which is assigned as the proton-transfer cysteine, forms an essential cysteine–SH interaction at Fe_D_ similar as observed in CbA5H. This interaction was determined to stabilize the I_2_ state leading to the conclusion that I_2_ (and therefore, the previously reported H_ox+1_ state)^6^ is H_inact_.

In this work, we established that there are several properties of CpIII H_ox+1_ that differentiate it from H_inact_ of CbA5H and DdH, which has been shown to be a result of SH interaction with the [2Fe] subsite, either in the form of sulfide binding or cysteine coordination. First, we reassigned the *E*_m_ (pH 8) value of H_ox+1_ to either −407 mV for H_ox+1_/H_ox_, or −418 mV for H_ox+1_/H_trans_ (Table 2). These values are more negative than the −357 mV (pH 7.4) value of the H_ox_/H_inact_ couple in CbA5H or −92 mV (pH 8) value for the H_inact_/H_trans_ couple in DdH.^33^ Second, unlike the H_inact_ states of CbA5H and DdH, the H_ox+1_ states of CpIII are observed in anaerobically prepared samples, or after reaction of resting H_ox+1_ with H_2_ (see Figures 2, 3, and S2). These conditions do not include extraneous sulfide,^2,30,32,36,38^ and our use of a biosynthetic maturation process to prepare CpIII alleviates H-cluster damage that can occur with chemically prepared diiron compounds.^6,13,21,25,26,31,41–48,57,60^ Apparently if the process has a low efficiency and incubation time, it can result in damage of the diiron compound and release of CN(−) (and CO) leading to the formation of H_inact_-like, and H_trans_-like­(CN-) bound states.^40^ Third, in CbA5H, the formation of H_inact_ requires the proton-transfer cysteine and was lost when exchanged to either aspartate or alanine,^36^ whereas, in CpIII the H_ox+1_ states are observed when this cysteine is exchanged to serine (Figure 5). Altogether, these details reveal distinguishing properties of the CpIII H_ox+1_ state that point to possible functional differences compared to H_inact_ of CbA5H and DdH^36,39^ but with possible similarity to the function of State 1 of *Tam*HydS.^34^ For the latter, possible aquo or hydroxide coordination at the [2Fe] subsite has been proposed,^34^ and while this may also be possible for CpIII, a detailed structural determination will be required to resolve the exact nature of the H_ox+1_ state.

## Conclusions

Overall, our studies on CpIII expand on the growing evidence that plasticity in the amino acids that form the secondary coordination sphere of the H-cluster functions to control oxidation states and catalysis. Future studies will address how the oxidation states of the H-cluster in CpIII culminate in the mechanism of H_2_ activation and catalysis. Resolving how the structural variations in CpIII support variation in H-cluster states and catalysis will add to the emerging understanding of how natural variation in cofactor sites, proton transfer residues, and conformational flexibility^2,6,15,17,34,39^ create profound differences in the catalytic properties and reactivity of hydrogenases to fulfill the diverse functions of these enzymes in metabolism.

## Supplementary Material





## Notes

Figure S1, Comparison of the AlphaFold/AlphaFill structural model and H-cluster environment of CpIII with the structure of CpI; Figure S2, FTIR spectra of CpIII treated with H_2_ and CO; Figure S3, variable-temperature spectra collected on H_2_-treated CpIII; Figure S4, plot of the midpoint potential analysis of potentiometric EPR spectra; Figure S5, variable-temperature EPR spectra collected on redox titration samples of CpIII; Figure S6, EPR simulations of potentiometric data collected at 20 K; Figure S7, FTIR spectra of AO native CpIII and CpIII C222S; Figure S8, EPR spectrum and simulation of auto-oxidized CpIII C222S. Table S1, FTIR signal assignments of CpIII and other [FeFe]-hydrogenases; Table S2, temperature and simulation parameters from EPR spectra of CpIII; Table S3, population analysis from simulation of EPR data (PDF)
CpIII AlphaFold/AlphaFill structural model manually refined to include the H-cluster and two F-clusters (PDB)

## Abbreviations

CpI: *Clostridium pasteurianum* [FeFe]-hydrogenase I
CpII: *Clostridium pasteurianum* [FeFe]-hydrogenase II
CpIII: *Clostridium pasteurianum* [FeFe]-hydrogenase III
DdH: *Desulfovibrio desulfuricans* [FeFe]-hydrogenase
CbA5H: Clostridium beijerinckii [FeFe]-hydrogenase
H-cluster: the [FeFe]-hydrogenase catalytic site cofactor
F-cluster: accessory iron–sulfur cluster
FC1: F-cluster 1
FC2: F-cluster 2
AO: auto-oxidized
H_inact_: fully oxidized, inactive H-cluster
H_ox+1_, H_ox+1_′: fully oxidized H-cluster
H_ox_: one-electron reduced H-cluster, [4Fe-4S]^2+^-[Fe^I^–Fe^II^]
H_trans_, and H_trans_-like: the one-electron reduced H-cluster; [4Fe-4S]^+^-[Fe^II^–Fe^II^]
H_red_: one-electron reduced H-cluster; [4Fe-4S]^+^-[Fe^II^–Fe^I^]
H_redH+_: one-electron reduced H-cluster, [4Fe-4S]^2+^-[Fe^I^–Fe^I^]
H_hyd_: one-electron reduced H-cluster with a hydride, [4Fe-4S]^+^-[Fe^II^–Fe^II^]-H^–^
H_sred_: super-reduced, or 2-electron reduced H-cluster, [4Fe-4S]^+^-[Fe^I^–Fe^I^]
H_ox_-CO: CO-inhibited state
CpIII C222S: CpIII with a serine-to-cysteine change at amino acid C222
FTIR: Fourier transform infrared spectroscopy
EPR: electron paramagnetic resonance spectroscopy
CO: carbon monoxide ligand
CN: cyanide ligand
Fe_D_: distal Fe atom of the H-cluster diiron subsite
NaDT: sodium dithionite
*T*_opt_: optimal temperature
mW: milliwatt
