# Supplementary material for: H‑cluster Intermediates and Catalytic Properties of Clostridium pasteurianum [FeFe]-Hydrogenase III
Source: Biochemistry. 2025 May 13;64(11):2455–66. doi: 10.1021/acs.biochem.5c00066 (PMC12138974; doi:10.1021/acs.biochem.5c00066)
Supplement: Supplementary file 1 [file bi5c00066_si_001.pdf]

Supporting Information

The H-cluster Intermediates and Catalytic Properties of *Clostridium pasteurianum* [FeFe]-hydrogenase III

Effie C. Kisgeropoulos[a‡], Michael W. Ratzloff[a‡], Ekaterina M Stroeva-Dahl[a], Sarah Hasan[a‡], Febin Varghese[b], Jacob H. Artz[a‡], John W. Peters[c], David W. Mulder[a*], Paul W. King[a,d*]

[a]Biosciences Center, National Renewable Energy Laboratory, Golden, CO 80401, United States. [b]Institute of Biological Chemistry, Washington State University, Pullman, WA 99163, United States. [c]Department of Chemistry and Biochemistry, University of Oklahoma, Norman, OK 73019, United States. [d]Renewable and Sustainable Energy Institute (RASEI), University of Colorado Boulder, Boulder, CO 80303, United States.

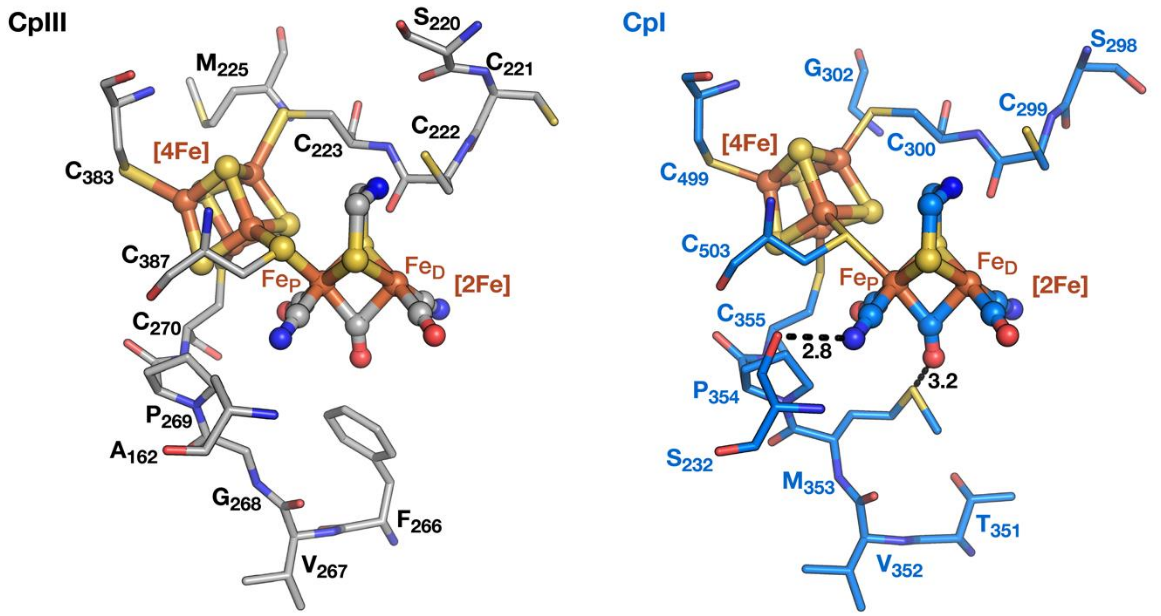

Figure S1. Comparison of the AlphaFold/AlphaFill structural model and H-cluster environment of CpIII (left) with the structure of CpI (right, PDB ID 3C8Y). Atom coloring scheme: Fe, rust; S, yellow-orange; C, gray (CpIII); C, marine (CpI); O, red; N, blue. Distances shown in Angstroms for CpI side chains of S232 and M353 with CN- and m-CO ligands, respectively of the diiron subsite. Abbreviations: [4Fe], [4Fe-4S] subsite of the H-cluster; [2Fe], diiron subsite of the H-cluster; FeD, distal Fe of [2Fe]; FeP, proximal Fe of [2Fe].

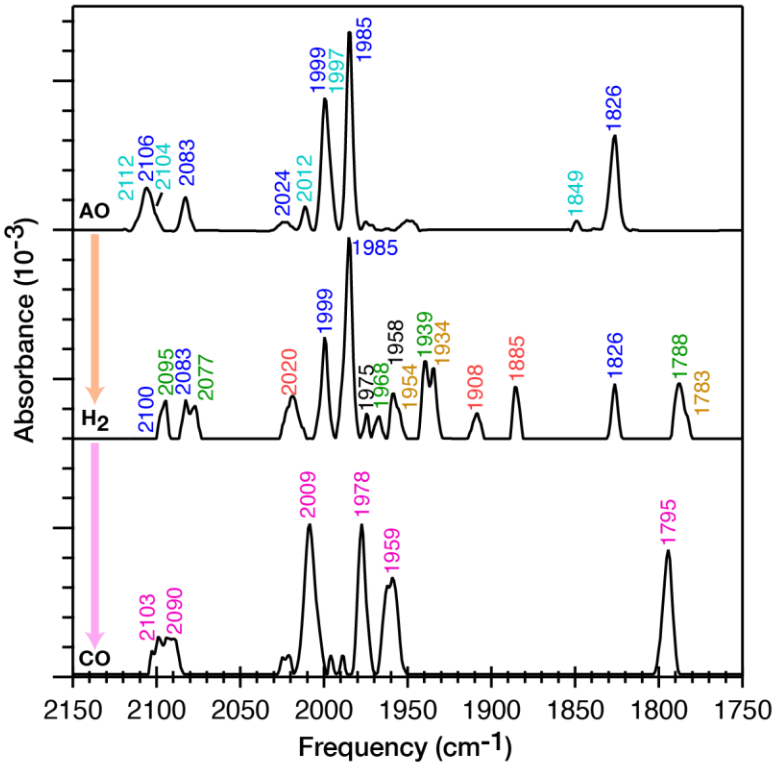

Figure S2. FTIR spectra of CpIII under various conditions. Auto-oxidized (AO, top panel). AO sample after equilibration under 1 atm of 100% H_2, pH 8 (middle panel). 100%H_2 treated sample exchanged into 100% CO (bottom panel). The νCN and νCO bands are grouped according to H-cluster oxidation states; H_{ox+1} (blue), H_{ox+1}' (cyan), H_{ox} (green), H_{trans}-like (black), H_{red} (orange), H_{redH+} (red), and $H_{ox\text{-}CO}$ (pink). The νCO and νCN bands assigned to H_{ox}-CO are not observed in the H_2, treated or AO CpIII WT spectra. IR spectra collected at 298 K. The 100% H_2-treated sample was prepared in a septum sealed conical vial by exchanging the headspace above an aliquot of AO with 100% H_2 by 10 vacuum/sparge cycles on a Schlenk line, and incubated overnight at 4 °C. Treatment with CO (Figure S2, bottom) was performed by sparging twice with 100% CO (each sparge was for 1 min followed by 10 min incubation on ice).

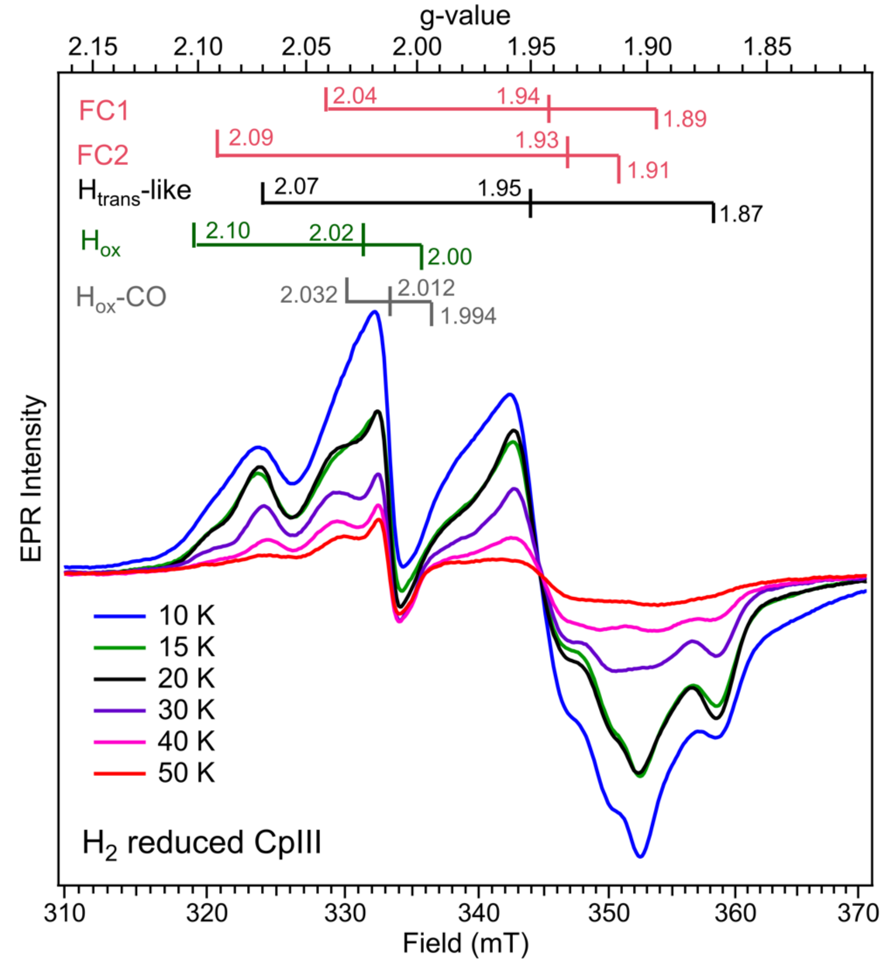

Figure S3. Variable-temperature EPR data collected on CpIIIWT reduced with 100% H_2 in the presence of sodium dithionite. Spectra are shown prior to intensity correction for the Curie law. Data collected using 1 mW of power. Top, *g*-values assignments for FC1 and FC2 (red), H_{trans}-like (black), Hox (green), and H_{ox}-CO (gray) species based on simulated *g*-values given in Table S2.

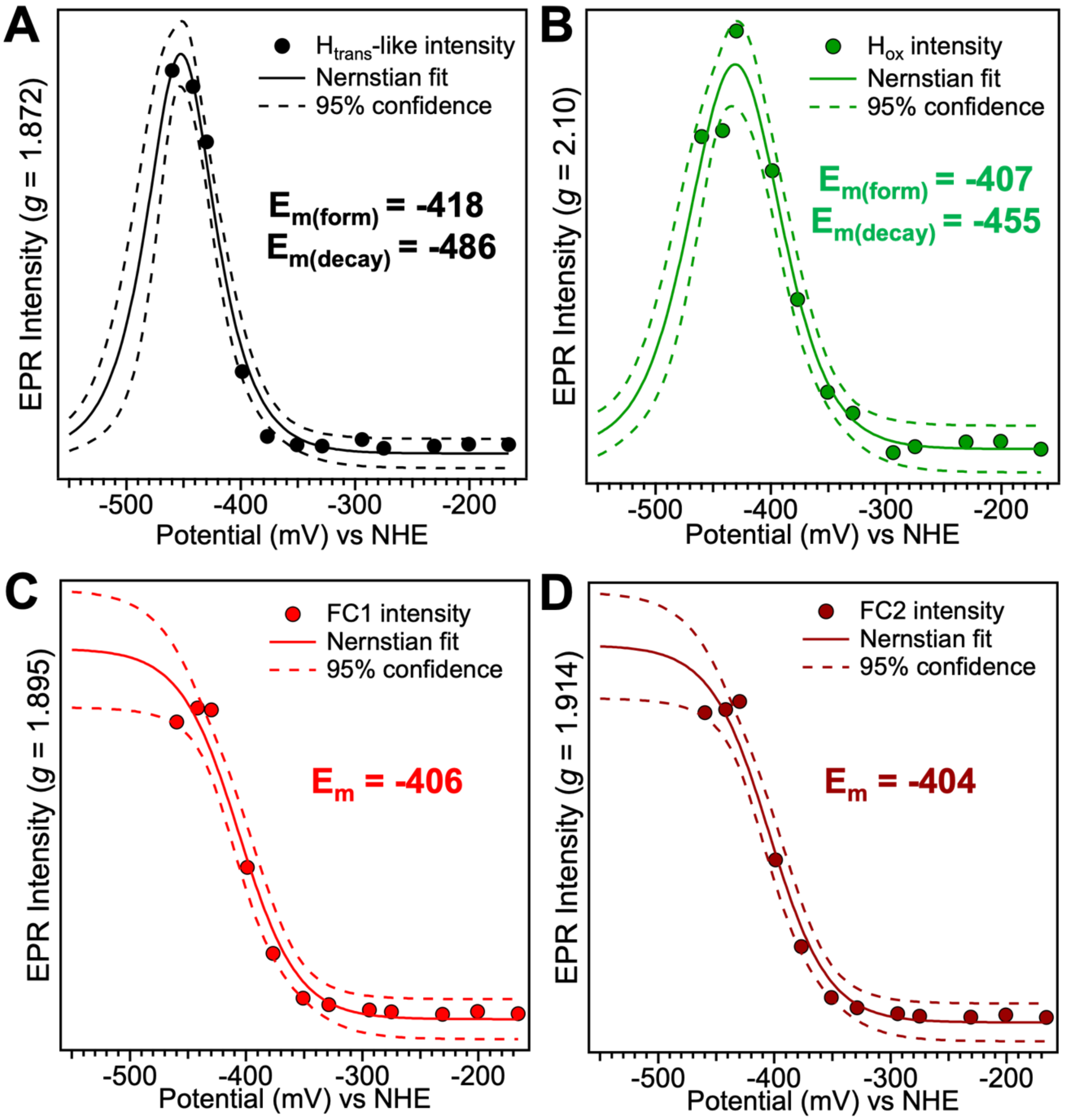

Figure S4. E_m analysis on the potentiometric response of H-cluster (**A**-**B**) and F-cluster (**C**-**D**) species in CpIII. Data for fitting obtained from potentiometric titration data collected using T = 15 K and P = 1 mW as described in reference [2]. Each species was monitored at the indicated feature and the resultant potential-dependent intensity changes (*circles*) fit to the Nernst equation using either a double (**A**-**B**; Equation S2) or single (**C**-**D**; Equation S1) form, as described in the Methods. The resultant fits (solid lines) produced E_m[8] values for the appearance of H_{trans}-like state (-418 mV), FC1 (-406 mV), and FC2 (-404 mV), and H_{ox} (-407 mV). The 95% confidence bands of the model are also shown overlaid as dashed lines. See Materials and Methods section *E_m analysis from 15 K titrations.*

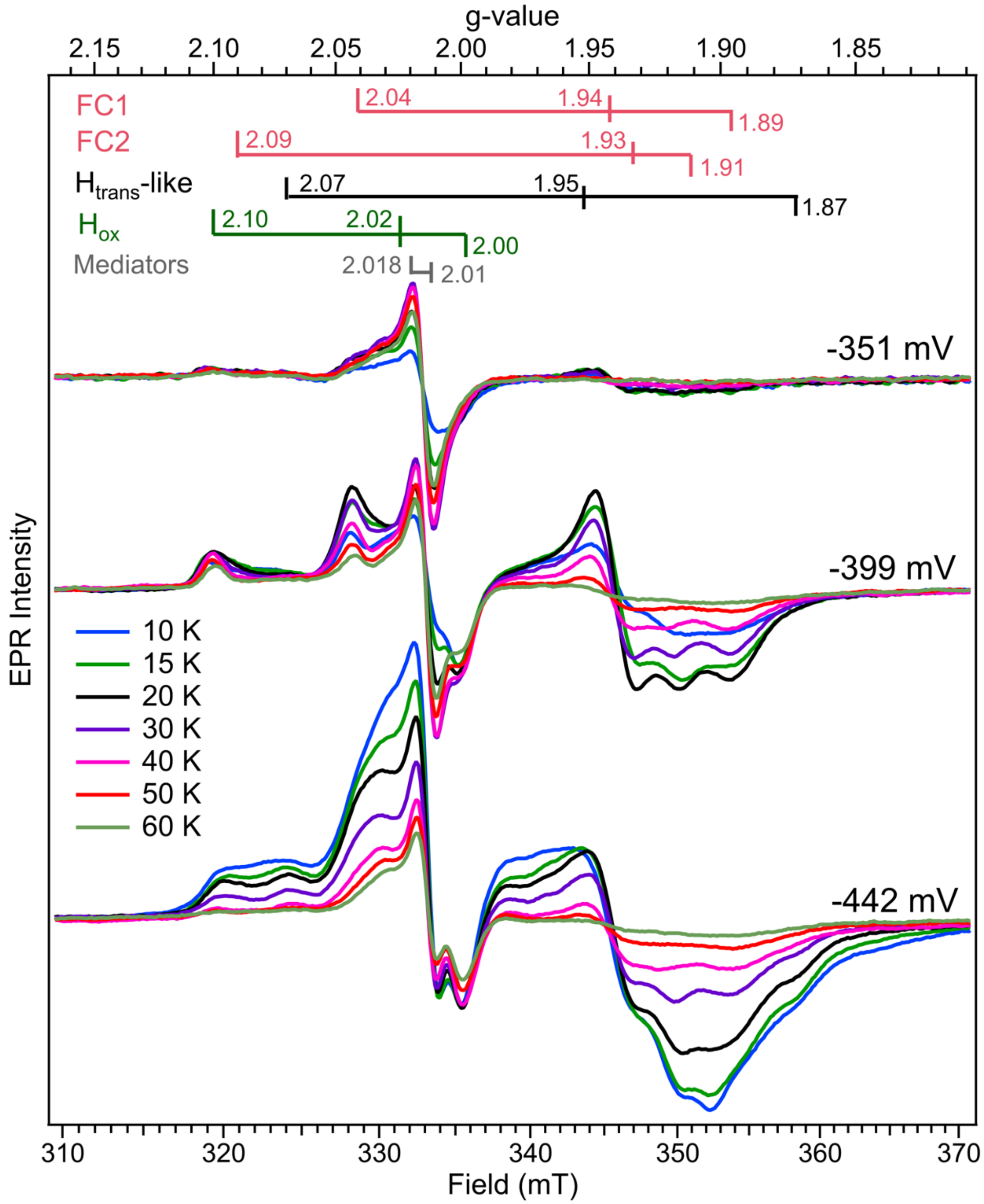

Figure S5. Variable-temperature EPR data collected on redox titration samples of CpIII poised at -351 mV (top), -399 mV (middle), and -442 mV vs NHE (bottom). Spectra are shown prior to intensity correction for the Curie law and on the same intensity scale. Data were collected using 1 mW of power. Analysis of the data corroborated the temperature-dependent properties of the H-cluster and FC signals determined from H_2 reduced data. **Top,** *g*-values assignments for FC1 and FC2 (red), H_{trans}-like (black), H_{ox} (green), and redox mediator (gray) species based on simulated *g*-values given in **Table S2**.

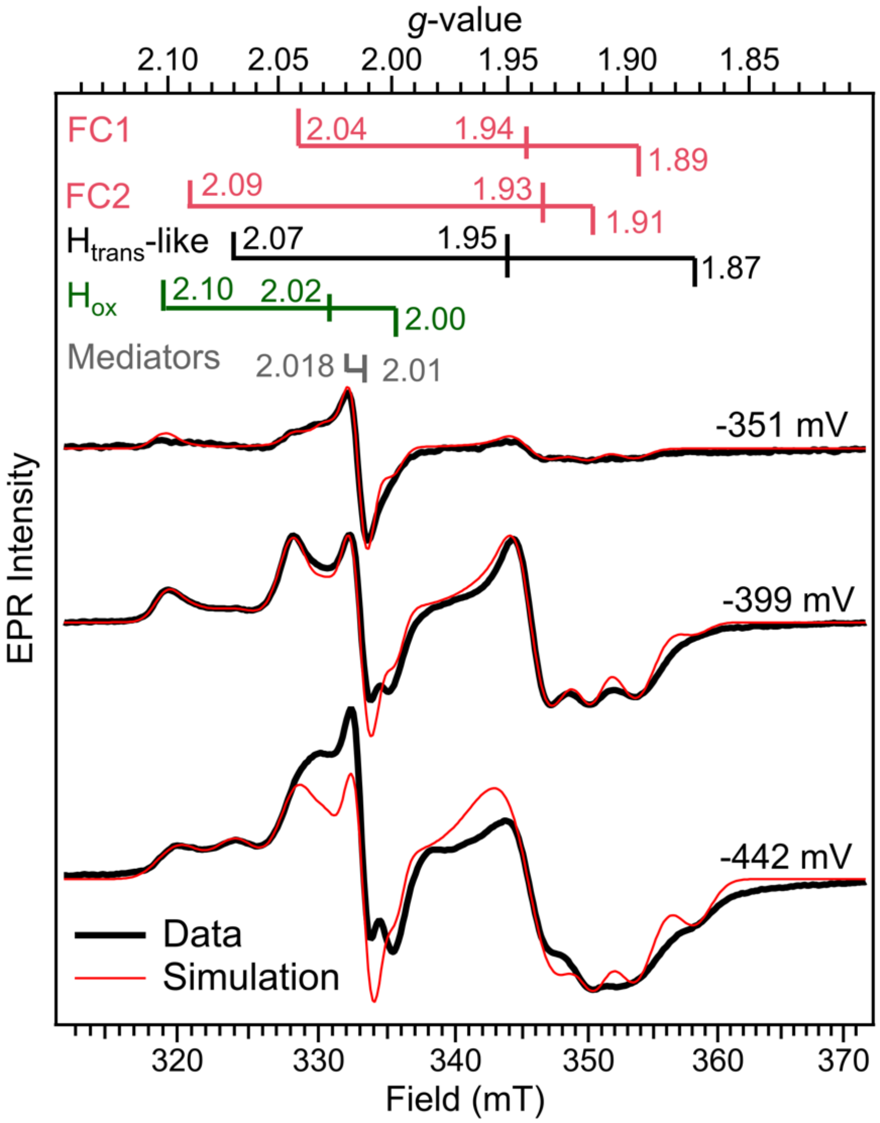

Figure S6. Potentiometric spectra of CpIII collected using X-band EPR. Samples (15 mg ml^{-1}) prepared in Artz et al.[1] and poised at -351, -399 or -442 mV vs NHE were used to collect spectra at T = 20 K and P = 1 mW (black traces). Spectral simulations (red traces) were performed using parameters listed in Table S2. Top, g-values assignments for FC1 and FC2 (red), H_{trans}-like (black), H_{ox} (green), and redox mediator (gray) species.

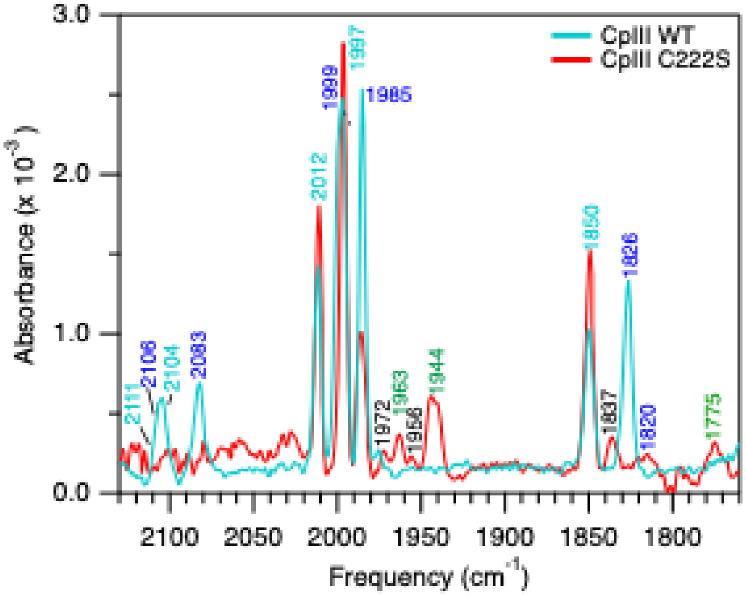

Figure S7. FTIR spectra of AO CpIII WT (cyan) and CpIII C222S (red) collected at 298 K. The νCO and νCN modes of H-cluster states; H_{ox+1} (blue), H_{ox+1}', (cyan), H_{ox} (green) and H_{trans}-like (black). Samples were anaerobically purified in buffer with sodium dithionite (2 mM, pH 8) and allowed to oxidize at 4 °C over a period of days or exchanged into dithionite free buffer prior to spectra collection.

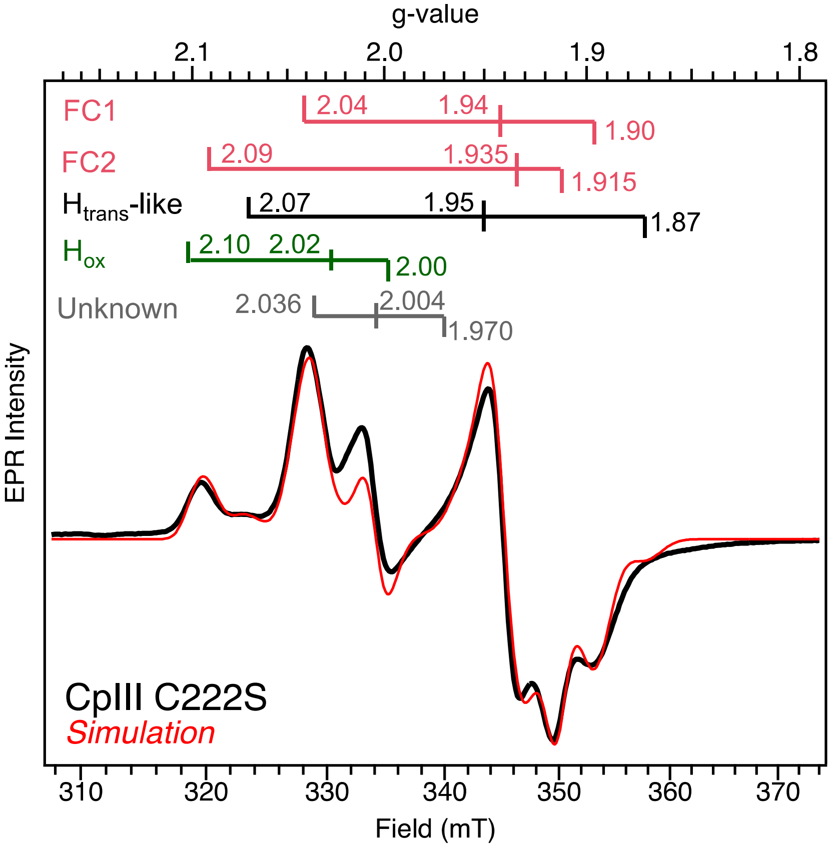

Figure S8. X-band EPR spectrum and simulation of auto-oxidized CpIII C222S. Signals attributed to FC1 and FC2 make up the majority of the spectrum along with contributions from Hox and H_{trans}-like species. An additional unknown signal with similarity to the CpIII H_{ox}-CO signal was also identified and simulated with a rhombic *g*-tensor at g = 2.036, 2.004, 1.970 (<10% relative contribution to the overall signal). Enzyme concentration at 7.8 mg ml^{-1} with data collected at T = 20 K, P = 1 mW. Assignments for the species and *g*-values are included at top (see Table S2 and S3).

Table S1. FTIR bands of CpIII H_{ox+1} states compared to H_{inact} and "State 1" observed in Group A and Group D [FeFe]-hydrogenases.

Enzyme	CN^-	t-CO	μ-CO	Name	Conditions	Ref
CpIII	2107, 2083	1999, 1985	1826	H_{ox+1}	Anaerobically oxidized (298 K; Fig. 2 and 5)	This work
CpIII	2102, 2083	1999, 1985	1823	H_{ox+1}	Anaerobically oxidized (195 K; Fig. 2)	This work
CpIII	2101, 2083	1999, 1985	1822	H_{ox+1}	Anaerobically oxidized (10 K; Fig. 2)	This work
CpIII	2106, 2083	2000, 1985	1826	H_{ox+1}	Oxidized with indigo disulfonate	[1]
CpIII	2107, 2083	1999, 1985	1826	H_{ox+1}	Thionine oxidized (298 K; Fig. 5)	This work
CpIII	2112, 2104	2012, 1997	1850	H_{ox+1}'	Anaerobically oxidized (298 K; Fig. 2 and Fig. 5)	This work
CpIII	2107, 2083	1999, 1985	1822	H_{ox+1}	Anaerobically oxidized (19 K; Fig. 5)	This work
CpIII	2112, 2104	2012	1847	H_{ox+1}'	Anaerobically oxidized (19 K; Fig. 5)	This work
CpIII C222S	2113, 2104	2012, 1998	1847	H_{ox+1}'	Anaerobically oxidized (15 K; Fig. 5)	This work
Ddh	N.R.	2008, 1983	1847		Purified in air	[2]
Ddh	2107, 2087	2007, 1983	1847		Purified in air	[3]
Ddh	2106, 2087	2007.5, 1983	1847.5	Air	Air	[4]
Ddh	2106, 2087	2007, 1983	1848	H_{inact}	Purified in air	[5]
Ddh	2106, 2086	2007, 1983	1847	H_{ox}^{Air}	Treated with hexaammineruthenium (III) chloride (HAR) → Na_2S → HAR	[6-7]
CbA5H	2107, 2080	2011, 1992	1840	H_{inact}	Oxidized with thionine or exposed to air	[8]
CbA5H	2107, 2080	2011, 1992	1840	H_{inact}	100% O_2	[9]
CbA5H	2107, 2080	2011, 1992	1840	H_{inact}	Exposed to O_2	[10]
TamHydS	2120, 2098	2016, 1999	1852	State 1	Exposed to air	[11]

Table S2. EPR temperature and simulation parameters.

Species	T_{opt}	Sample	*g*-values	*g*-value strains	Figure
H_{ox}	40 – 50 K	-351 mV	2.100, 2.024, 1.998	0.013, 0.011, 0.010	S6
		-399 mV		0.013, 0.011, 0.009	S6
		-442 mV		0.018, 0.010, 0.008	S6
		H_2/DT red.		0.012, 0.011, 0.011	4
		H_2 red.		0.012, 0.015, 0.011	4
	[a]N.D.	AO C222S		0.012, 0.014, 0.017	S8
H_{trans}-like	20 K	-351 mV	[a]N.D.	[a]N.D.	S6
		-399 mV	2.070, 1.952, 1.872	0.020, 0.020, 0.014	S6
		-442 mV	2.0695, 1.952, 1.872	0.022, 0.022, 0.016	S6
		H_2/DT red.	2.072, 1.952, 1.8715	0.020, 0.019, 0.014	4
		H_2 red.	2.073, 1.952, 1.872	0.020, 0.019, 0.015	4
	[a]N.D.	AO C222S	2.073, 1.952, 1.872	0.020, 0.019, 0.018	S8
FC1	20 – 30 K	-351 mV	2.041, 1.942, 1.895	0.011, 0.012, 0.014	S6
		-399 mV	2.043, 1.941, 1.895	0.014, 0.014, 0.0175	S6
		-442 mV	2.041, 1.941, 1.896	0.018, 0.020, 0.0185	S6
		H_2/DT red.	2.041, 1.942, 1.896	0.020, 0.022, 0.016	4
		H_2 red.	2.041, 1.942, 1.896	0.020, 0.018, 0.017	4
	[a]N.D.	AO C222S	2.043, 1.942, 1.896	0.016, 0.013, 0.018	S8
FC2	20 – 30 K	-351 mV	2.091, 1.935, 1.914	0.018, 0.012, 0.012	S6
		-399 mV	2.091, 1.935, 1.914	0.020, 0.014, 0.012	S6
		-442 mV	2.091, 1.935, 1.9135	0.018, 0.020, 0.013	S6
		H_2/DT red.	2.091, 1.934, 1.912	0.018, 0.022, 0.014	4
		H_2 red.	2.091, 1.934, 1.912	0.018, 0.017, 0.015	4
	[a]N.D.	AO C222S	2.093, 1.935, 1.915	0.015, 0.014, 0.012	S8
[b]H_{ox}-CO	[a]N.D.	CO treated	2.0315, 2.012, 1.9935	0.0135, 0.010, 0.013	4
		H_2/DT red.	2.0315, 2.012, 1.9935	0.0135, 0.010, 0.013	4
		H_2 red.	2.0315, 2.012, 1.9935	0.0142, 0.008, 0.030	4
[c]Unknown	[a]N.D.	AO C222S	2.036, 2.004, 1.970	0.012, 0.012, 0.025	S8
Mediators	[a]N.D.	-351 mV	2.018, 2.012, 2.012	[d]70, 40, 40	S6
		-399 mV	2.018, 2.011, 2.011	[d]50, 49, 49	S6
		-442 mV	2.018, 2.010, 2.010	[d]50, 49, 49	S6

[a]N.D. for not determined.

[b]In the sample treated with CO a second isotropic-type signal (*g* = 2.017 2.008 2.008; *g*-strains = 0.012 0.01 0.01) was also resolved (see **Methods**).

[c]In the AO sample of CpIII C222S additional signal intensity present in the middle of the spectrum is attributed to an unknown (unassigned) species. This intensity was fit using a rhombic *g*-tensor and included in the simulation (at a relative contribution of

<10%; **Table S3**). While the *g*-values of this species have some similarity to those of the CpIII H_{ox}-CO species they display significantly more anisotropy.

[d]For the mediator signal, broadening of the simulated spectrum was conducted using the Easyspin HStrain function rather than gStrain given the physical origin of the signal as a radical species rather than a spin-delocalized FeS cluster.

Table S3. H-cluster and FC populations[a] determined from EPR spectra under various treatments.

Species	[b]H_2/DT reduced	[b]H_2 reduced	[b]H_2 then CO	[c]-351 mV	[c]-399 mV	[c]-442 mV	AO C222S
H_{ox}	1.7%	3.0%	–	35%	10.1%	6.2%	3.6%
H_{trans}-like	48.1%	44.7%	–	–	11.8%	31.0%	12.9%
FC1	33.9%	28.3%	–	35%	55.5%	45.5%	47.9%
FC2	14.1%	16.4%	–	20%	20.2%	15.5%	26.8%
$H_{ox\text{-}CO}$	1.7%	6.7%	73.3%	–	–	–	–
Unknown	–	–	–	–	–	–	[d]8.8%
Total simulated intensity	1.768 (100%)	1.342 (100%)	0.150 (100%)	0.100 (100%)	0.594 (100%)	0.967 (100%)	1.94 (100%)

[a]Population of each species is reported as its % of the total simulated intensity, determined from simulation of EPR spectra in **Figures 4, S6,** and **S8**, using parameters given in **Table S2**. Dashes indicate the species was not identified or resolvable in spectrum.

[b]The additional isotropic-type signal identified in treatment with CO was included at 0.5% (H_2/DT reduced), 0.9% (H_2 reduced), and 26.7% (CO treated).

[c]Signal from the redox mediators was also included in each simulation at 10% (-351 mV), 2.4% (-399 mV), and 1.8% (-442 mV), of the total simulated intensity.

[d]This number represents an additional (unknown) species in the auto-oxidized spectrum of the CpIII C222S variant with *g* = [2.036, 2.004 1.970] (see **Table S2**).

References

1. Artz, J. H.; Zadvornyy, O. A.; Mulder, D. W.; Keable, S. M.; Cohen, A. E.; Ratzloff, M. W.; Williams, S. G.; Ginovska, B.; Kumar, N.; Song, J.; McPhillips, S. E.; Davidson, C. M.; Lyubimov, A. Y.; Pence, N.; Schut, G. J.; Jones, A. K.; Soltis, S. M.; Adams, M. W. W.; Raugei, S.; King, P. W.; Peters, J. W., Tuning Catalytic Bias of Hydrogen Gas Producing Hydrogenases. Journal of the American Chemical Society 2020, 142 (3), 1227-1235.
2. Van Der Spek, T. M.; Arendsen, A. F.; Happe, R. P.; Yun, S.; Bagley, K. A.; Stufkens, D. J.; Hagen, W. R.; Albracht, S. P. J., Similarities in the Architecture of the Active Sites of Ni-Hydrogenases and Fe-Hydrogenases Detected by Means of Infrared Spectroscopy. European Journal of Biochemistry 1996, 237 (3), 629-634.
3. Nicolet, Y.; de Lacey, A. L.; Vernède, X.; Fernandez, V. M.; Hatchikian, E. C.; Fontecilla-Camps, J. C., Crystallographic and FTIR Spectroscopic Evidence of Changes in Fe Coordination Upon Reduction of the Active Site of the Fe-Only Hydrogenase from Desulfovibrio desulfuricans. Journal of the American Chemical Society 2001, 123 (8), 1596-1601.
4. Pierik, A. J.; Hulstein, M.; Hagen, W. R.; Albracht, S. P. J., A low-spin iron with CN and CO as intrinsic ligands forms the core of the active site in [Fe]-hydrogenases. European Journal of Biochemistry 1998, 258 (2), 572-578.
5. Roseboom, W.; De Lacey, A. L.; Fernandez, V. M.; Hatchikian, E. C.; Albracht, S. P. J., The active site of the [FeFe]-hydrogenase from Desulfovibrio desulfuricans. II. Redox properties, light sensitivity and CO-ligand exchange as observed by infrared spectroscopy. JBIC Journal of Biological Inorganic Chemistry 2006, 11 (1), 102-118.
6. Rodríguez-Maciá, P.; Reijerse, E. J.; van Gastel, M.; DeBeer, S.; Lubitz, W.; Rüdiger, O.; Birrell, J. A., Sulfide Protects [FeFe] Hydrogenases From O2. Journal of the American Chemical Society 2018, 140 (30), 9346-9350.
7. Rodríguez-Maciá, P.; Galle, L. M.; Bjornsson, R.; Lorent, C.; Zebger, I.; Yoda, Y.; Cramer, S. P.; DeBeer, S.; Span, I.; Birrell, J. A., Caught in the Hinact: Crystal Structure and Spectroscopy Reveal a Sulfur Bound to the Active Site of an O2-stable State of [FeFe] Hydrogenase. Angewandte Chemie International Edition 2020, 59 (38), 16786-16794.
8. Morra, S.; Arizzi, M.; Valetti, F.; Gilardi, G., Oxygen Stability in the New [FeFe]-Hydrogenase from Clostridium beijerinckii SM10 (CbA5H). Biochemistry 2016, 55 (42), 5897-5900.
9. Corrigan, P. S.; Tirsch, J. L.; Silakov, A., Investigation of the Unusual Ability of the [FeFe] Hydrogenase from Clostridium beijerinckii to Access an O2-Protected State. Journal of the American Chemical Society 2020, 142 (28), 12409-12419.
10. Winkler, M.; Duan, J.; Rutz, A.; Felbek, C.; Scholtysek, L.; Lampret, O.; Jaenecke, J.; Apfel, U.-P.; Gilardi, G.; Valetti, F.; Fourmond, V.; Hofmann, E.; Léger, C.; Happe, T., A safety cap protects hydrogenase from oxygen attack. Nature Communications 2021, 12 (1), 756.
11. Cabotaje, P. R.; Walter, K.; Zamader, A.; Huang, P.; Ho, F.; Land, H.; Senger, M.; Berggren, G., Probing Substrate Transport Effects on Enzymatic Hydrogen Catalysis: An Alternative Proton Transfer Pathway in Putatively Sensory [FeFe] Hydrogenase. ACS Catalysis 2023, 13 (15), 10435-10446.
